# Protective Effect of *Bruguiera gymnorrhiza* (L.) Lam. Fruit on Dextran Sulfate Sodium-Induced Ulcerative Colitis in Mice: Role of Keap1/Nrf2 Pathway and Gut Microbiota

**DOI:** 10.3389/fphar.2019.01602

**Published:** 2020-02-03

**Authors:** Yinsi Lin, Xinghan Zheng, Jinfen Chen, Dandan Luo, Jianhui Xie, Ziren Su, Xiaoqi Huang, Xiaoqing Yi, Long Wei, Jian Cai, Zhanghua Sun

**Affiliations:** ^1^ Guangdong Provincial Key Laboratory of New Drug Development and Research of Chinese Medicine, Mathematical Engineering Academy of Chinese Medicine, Guangzhou University of Chinese Medicine, Guangzhou, China; ^2^ Guangdong Provincial Key Laboratory of Clinical Research on Traditional Chinese Medicine Syndrome, The Second Affiliated Hospital, Guangzhou University of Chinese Medicine, Guangzhou, China; ^3^ Guangdong Provincial Key Laboratory of Silviculture, Protection and Utilization, Guangzhou, China; ^4^ Guangdong Academy of Forestry, Guangzhou, China

**Keywords:** *Bruguiera gymnorrhiza*, ulcerative colitis, pinitol, Keap1/Nrf2, gut microbiota

## Abstract

*Bruguiera gymnorrhiza* (BG), a medicinal mangrove, and its fruit (a food material) (BGF), have traditionally been used to treat diarrhea (also known as ulcerative colitis) in folk medicine. However, the mechanism of action against colitis remains ambiguous. This study aimed to investigate the potential efficacy and mechanism of BGF on experimental colitis. Colitis was induced by oral intake of dextran sulfate sodium (DSS) and treated with aqueous extract of BGF (25, 50 and 100 mg/kg) for a week. The Disease Activity Index (DAI), colon length, and histological changes of colon were analyzed. The inflammatory and oxidative stress status was explored. The protein expression of Nrf2 and Keap1 in the colon was detected by Western blotting. The mRNA expression of Nrf2 downstream genes (*GCLC*, *GCLM*, *HO-1* and *NQO1*) was determined by RT-PCR. Furthermore, the effect on intestinal flora was analyzed. Results indicated that BGF was rich in pinitol, and showed strong antioxidative activity *in vitro*. Compared with the DSS model, BGF effectively reduced the body weight loss and DAI, restored the colon length, repaired colonic pathological variations, and decreased the histological scores, which was superior to salicylazosulfapyridine (SASP) with smaller dosage. Moreover, BGF not only abated the levels of MDA and inflammatory mediators (TNF-α, IL-6, IL-1β, and IFN-γ), increased the level of IL-10, but also prevented the depletion of SOD and GSH. BGF upregulated the protein level of nuclear Nrf2 and mRNA levels of *GCLC*, *GCLM*, *HO-1* and *NQO1*, while significantly inhibited the protein expression of Keap1 and cytosolic Nrf2. Besides, BGF promoted the growth of probiotics (*Bifidobacterium*, *Anaerotruncus*, and *Lactobacillus*) in the gut, and inhibited the colonization of pathogenic bacteria (*Bacteroides* and *Streptococcus*), which contributed to the maintenance of intestinal homeostasis. BGF possessed protective effect against DSS-induced colitis. The potential mechanism of BGF may involve the amelioration of inflammatory and oxidative status, activation of Keap1/Nrf2 signaling pathway, and maintenance of micro-ecological balance of the host. This study provides experimental evidence for the traditional application of BGF in the treatment of diarrhea, and indicates that BGF may be a promising candidate against colitis.

## Introduction

Ulcerative colitis (UC), a chronic inﬂammatory disease which occurs in the rectum and colon, belongs to inflammatory bowel disease (IBD). It is a kind of chronic intestinal inflammation caused by multiple causes, like genetic factors, lifestyle, and disruption of the microbial ecosystem in colon (Eisenstein, 2018). UC clinically manifests chronic diarrhea, durative or repeatable mucous, abscess, and blood defecate with symptoms of different degrees. Notably, its protracted disease process generally results in complications, even colorectal cancer. Besides, its morbidity has been trending upward and younger (Trivedi et al., 2018). Although patients can take medicine to control UC, such as sulfasalazine (SASP) and mesalazine, it often results in various side effects, such as fever, vomit, and acute pancreatitis (Russo et al., 2014). Therefore, ulcerative colitis has been regarded as one of the cureless diseases by World Health Organization, jeopardizing health as well as quality of life (Kaplan, 2015). It is inevitable to search safe alternatives for the therapy of UC.

UC is closely associated with the imbalance of oxidative stress (Nikkhah-Bodaghi et al., 2019). When UC is induced in mice, excessive amounts of reactive oxygen species (ROS) are produced by activated macrophages and neutrophils in the inflamed intestine. Moreover, an imbalance of oxidative stress and the limitation of the intestinal antioxidant defense system have been observed in colitis (Yasukawa et al., 2019). NF-E2-related factor 2 (Nrf2), a key factor in oxidative stress response of cells regulated by Kelch-like ECH-associated protein 1 (Keap1), moderates the expression of antioxidant proteins and phase II detoxification enzymes (Kitakaze et al., 2019). UC is connected with the Keap1/Nrf2 signaling pathway, and influences Nrf2, Keap1, and Nrf2-related target genes and their expression (Lu et al., 2016). Glutamate-cysteine ligase catalytic subunit (GCLC), glutamate-cysteine ligase modifier subunit (GCLM), hemeoxygenase-1 (HO-1) and NAD(P)H quinone dehydrogenase 1 (NQO1) belong to the Nrf2 downstream antioxidant enzymes, which are associated with treatment of UC (Song et al., 2019). Therefore, activation of Keap1/Nrf2 signaling pathway could be a promising therapeutic approach for UC.

Abnormal intestinal mucosal immunity induced by intestinal flora disorder, is one of the important causes of UC (Liu et al., 2019b). Under physiological conditions, the intestinal microecology remains in a state of dynamic equilibrium. When intestinal flora is out of balance, harmful bacteria grow excessively, which leads to the occurrence of colon diseases. It has been revealed that probiotics are closely related to human health, especially the intestinal barrier (Asto et al., 2019). Therefore, the regulation of intestinal microecology is conducive to the control of development of UC.

There is increasing interest in natural medicine as a source of alternative therapy for UC (Ma et al., 2018; Lopes de Oliveira et al., 2019). *Bruguiera gymnorrhiza* (BG), a dominant specie of mangroves and a traditional medicinal plant, has attracted increasing attention in recent years. BG has been found to be endowed with appreciable biological activities, such as antioxidation, anti-plasmodium and anticancer (Sarkar et al., 2013; Sudirman et al., 2014). As a food with starch, *B. gymnorrhiza* fruit (BGF) are sliced, soaked to flush out the tannins and then ground to a paste, which can be an ingredient for pastry (Bandaranayake and Marshes, 1998). In addition, BGF has been commonly used to treat chronic diarrhea for many years (Mahmud et al., 2017), which are also known as ulcerative colitis according to TCM theory. However, current investigations have been mainly focused on its anticancer and anti-diabetic effects, seldom endeavor has been dedicated to illuminating its traditional application like diarrhea.

Since BGF has long been used in traditional folk medicine for the treatment of chronic diarrhea, and given the proceeding promising findings on its antioxidant activity and the vital role of oxidative equilibrium and intestinal flora in the pathogenesis of UC, it is therefore logical to hypothesize that BGF may exert protective effect against UC by favorably regulating the Keap1/Nrf2-mediated oxidative status and intestinal flora. To experimentally test this hypothesis, in the present study, we endeavored to explore the potential effects of BGF on a murine model of dextran sulfate sodium (DSS)-induced UC and unravel the mechanism of action.

## Materials and Methods

### Materials and Reagents

*B. gymnorrhiza* (L.) Lam. was provided by Nansha Wetland Park (Guangzhou, Guangdong, China), and was authenticated by one of our authors, Prof. Ziren Su of Guangzhou university of Chinese medicine, where a voucher specimen (Voucher 18-06-23) was deposited. HPLC grade methanol was purchased from Merck (Darmstadt, Germany). 1,1-Diphenyl-2-picryl-hydrazyl (DPPH, CAS:1898-66-4) was purchased from Shanghai Macklin Biochemical Co., Ltd. Dextran sulfate sodium (DSS) was bought from MP Biomedicals (molecular weight: 36,000~50,000, Canada). SASP was purchased from Shanghai Xinyi Tianping Pharmaceutical Co. Ltd (Shanghai, China). Myeloperoxidase (MPO) assay kit was obtained from Jiancheng Biotechnology Company (Nanjing, Jiangsu, China). MDA, SOD, and GSH assay kits were purchased from Jiancheng Biotechnology Company (Nanjing, Jiangsu, China). The enzyme-linked immunosorbent assay (ELISA) kits for TNF-α, IL-6, IL-1β, IFN-γ, IL-10, iNOS, and COX-2 were the products of Shanghai MLBIO Biotechnology Co. Ltd (Shanghai, China). The primary and secondary antibodies used in this study were purchased from Affinity Biosciences (OH, USA). The cDNAs for *GCLC*, *GCLM*, *HO-1* and *NQO1* were amplified by PCR with gene specific primers (Sangon Biotech Co. Ltd, Shanghai, China). Other chemicals used were of analytical grade or chromatographic grade.

### Preparation of the Plant Extracts

The powder of *B. gymnorrhiza* fruit (500 g) was heated to reflux for 2.5 h with 10 times volume water. The extraction was repeated three times. The extracting solution was filtered to remove the residue, and then was concentrated by rotary evaporator. Moreover, the concentrated solution was freeze-dried under vacuum. The lyophilized powder of BGF (77.75 g) was kept at 4 °C in the refrigerator for further assay.

### Qualitative and Quantitative Analysis of BGF Aqueous Extract

Before pharmacological evaluation, the main phytochemical components of BGF were analyzed by LC-MS-IT-TOF, NMR, and HPLC. The tentative identiﬁcation of the extract components was based on molecular weights, MS^3^ fragmentation, as well as literature data. The UPLC system consisted of a Shimadzu LC-20A instrument (Japan) equipped with two quaternary pumps (LC-20AD) and an automatic injector (SIL-20A). The separation was performed on a Shimadzu Shim-pack GISS C_18_ column (1.9 μm, 100 × 2.1 mm) with a flow rate of 0.2 mL/min. For the mobile phase, methanol (solvent A) and 0.1% formic acid (solvent B) were used. Gradient elution began with 10% solvent A and 90% solvent B. Elution solvents were changed to 50% A for 15 min. The MS analysis was operated in both positive and negative modes, and the scan range was set at *m/z* 100–2,000. BGF sample solutions were diluted with water, and then ﬁltered through a membrane ﬁlter (0.22 μm pore size). Two μL of the sample was injected into the UPLC instrument. Data were analyzed with Shimadzu LC-solution software (Kyoto, Japan) and ACD/Labs software (Canada).

To further identify the major composition of BGF, One-dimensional (1D) nuclear magnetic resonance (NMR) experiment was performed in deuterated dimethyl sulfoxide (DMSO-*d_6_*), using the NMR Bruker 400 NMR spectrometer. Solutions for NMR spectra were prepared by dissolving about 40 mg BGF in 0.5 mL DMSO-*d_6_*. Scan number of ^13^C NMR was set to 256. The ^1^H and ^13^C NMR chemical shifts were speciﬁed in ppm. Data were analyzed with MestReNova 12.0 software (Mestrelab Research Company, Spain).

Quantitative analysis of pinitol in BGF aqueous extract was performed on a Shimadzu LC-20A HPLC system equipped with an Alltech 3300 ELSD detector, using a Cosmosil Sugar-D column (4.6 × 250 mm, 5 µm) at a column temperature of 30 °C. The ﬂow rate and injection volume were 1 mL/min and 5 µL, respectively. The acetonitrile-water (20:80, v: v) system was employed as the mobile phase for 15 min for quantitative determination of standard compound (pinitol). The standard compound was dissolved in water. The temperature of the nebulizer was set at 55°C with the nitrogen as developing solvent.

### Antioxidant Activity of BGF *in Vitro*

DPPH free radical Scavenging assay. DPPH test is a method widely used to determine the antioxidative efficiency of a substance. The radical scavenging activity and the reducing power of BGF were determined according to the method previously reported (Liu et al., 2019a). Briefly, 2.0 mL DPPH solution (0.08 mg/mL) in methanol was added to 1 mL plant extract (20, 40, 60, 80, 100 and 120 µg/mL). After incubation for 30 min at room temperature, the absorbance was measured against a blank containing sample and methanol using a UV-Vis spectrophotometer at 517 nm. Ascorbic acid (Vc) was used as a positive control. The inhibition curve was plotted and the IC_50_ values were determined. The percentage of radical scavenging activity was calculated as:

$$\text{DPPH radical scavenging activity }\left(\%\right)\;=\;\left(1-\frac{{\text{A}}_{0}-{\text{A}}_{1}}{{\text{A}}_{2}}\right)*100\%$$

Where A_0_ is the absorbance of the sample and DPPH solution, A_1_ is the absorbance of sample solution with methanol, and A_2_ is the absorbance of a mixture of DPPH solution and distilled water.

Reducing power assay. The absorbance of the reducing power of BGF was detected at 700 nm. Ascorbic acid was used as a positive control. 2.5 mL potassium ferricyanide K_3_[Fe(CN)_6_] (1.0%, w/v) and 2.5 mL phosphate buffer (0.2 M, pH 6.6) were mixed with different concentrations of BGF and incubated at 50 °C for 20 min. In addition, 2.5 mL trichloroacetic acid (10%, w/v) was added to the mixture for centrifugation at 1,500 g for 10 min. The supernatant (2.5 mL) was mixed with 0.5 mL ferric trichloride (1.0%, w/v) and 2.5 mL distilled water. The absorbance was detected at 700 nm.

### Experimental Animals

Male BALB/c mice (20-25 g, seven-eight weeks), were obtained from the Laboratory Animal Services Center, Guangzhou University of Chinese Medicine (Guangzhou, China). Mice received standard food and sterilized water *ad libitum* under controlled conditions where there was constant temperature (20–25°C) and humidity (60–70%) with a 12-h light/dark cycle. This study was performed based on the National Institutes of Health guide for the care and use of laboratory animals (NIH Publications No. 8023). The experimental protocols followed the Animal Ethics Committee of Guangzhou University of Chinese Medicine.

### Induction of Acute Colitis Model and Treatment

Three dosages of BGF (25, 50 and 100 mg/kg) were selected according to our prior trial. A total of 70 male mice in this experiment were randomly divided into six groups: normal group (*n* = 10), DSS model group (*n* = 14), SASP group (200 mg/kg, *n* = 10), BGL group (25 mg/kg, *n* = 10), BGM group (50 mg/kg, *n* = 10) and BGH group (100 mg/kg, *n* = 16). Experimental colitis was induced by oral administration of 3% DSS (w/v) in drinking water for 7 days following previous regime (He et al., 2019). Normal group received drinking water every day for a week, and five other groups were given 3% DSS once daily for a week. Simultaneously, mice of SASP and three BGF groups were orally administered with SASP (200 mg/kg) or aqueous extract of BGF (25, 50 and 100 mg/kg), respectively, while the normal and DSS model groups received normal saline (0.1 ml/10 g). Experimental schedule was shown in **Figure 2A**. The body weight, stool consistency, rectal bleeding, food intake, water intake, and general appearance of all mice were evaluated daily.

### Disease Activity Index (DAI) Assessment

Body weight, feces status, and bloody stools of all mice were observed and recorded every day (Sann et al., 2013). Changes of body weight were calculated as the percent difference between the original body weight and the body weight on each day, and expressed as percentage loss of the baseline body weight. The DAI scores were assessed on the 7^th^ day (Qu et al., 2017). Bleeding score in feces was analyzed using an occult blood kit according to the below criteria (**Table 1**).

**Table 1:** The criteria of DAI.

Body weight loss	Stool consistency	Occult blood or bloody stool	Score
No change	Normal	Negative	0
1–5%	Loose stool	Negative	1
5–10%	Loose stool	Occult blood positive	2
10–20%	Diarrhea	Occult blood positive	3
≥20%	Diarrhea	Gross hematochezia	4

### Evaluation of Histological Change

At the end of the experiment, mice were sacriﬁced by cervical dislocation. The colons were quickly removed, rinsed with sterile and cold physiological saline, and moved for the measurement of the length of colon. The macroscopic evaluation for colons was also performed. In addition, the colon tissue was fixed in paraffin wax, sectioned, and stained with H&E following routine manner. Assessment was carried out following the criteria of histological score (**Table 2**) previously reported (Erben et al., 2014).

**Table 2:** The criteria of histological score.

Categories	Feature	Score
Architectural changes	No change	0
	Mild abnormality in mucosa	1
	Moderate diffuse or multifocal abnormalities in submucosa	2
	Severe diffuse or multifocal abnormalities	3
	Peritonitis	4
Inflammatory infiltrate	No change	0
	Mild but unequivocal increase	1
	Moderate change	2
	Marked change	3
	Severe increase	4
Lamina propria leukocytes	No changes	0
	Mild increase	1
	Moderate increase	2
	Marked increase	3
	Severe increase	4
Intraepithelial neutrophils	No changes	0
	25%	1
	50%	2
	75%	3
	100%	4
Erosion or ulceration	No change	0
	Erosion focally stripped	1
	Marked erosion	2
	Severe erosion	3
	Ulceration or granulation tissue	4
Crypt destruction	No change	0
	Blunted crypts	1
	Marked attenuation	2
	Crypt necrosis	3
	No architecture	4

### Myeloperoxidase (MPO) Assessment

Colons were homogenized in 0.1 M ice-cold phosphate buffer (pH 7.4), the homogenates were centrifuged at 10,000 g for 15 min at 4 °C, and the supernatant was collected for biochemical estimation. MPO activity, a key indicator of neutrophil infiltration, was measured with a MPO assay kit in accordance with the kit instructions. Experiments were performed three times.

### Measurement of Inflammatory Cytokines

The levels of inﬂammatory mediators (TNF-α, IL-6, IL-1β, IFN-γ, and IL-10) in the colon tissues were measured by valid enzyme-linked immunosorbent assay (ELISA) kits (Shanghai MLBIO Biotechnology Co. Ltd, China) in accordance with the kit instructions.

### Detection of Oxidative Stress Markers

Colons were added with saline ice, and then homogenized to obtain 10% colon homogenate. The homogenate was centrifuged for 10 min (5,000 g, 4 °C) to obtain the supernatant. Superoxide dismutase (SOD), glutathione (GSH) and malondialdehyde (MDA) levels were measured *via* corresponding assay kits from Jiancheng Bioengineering Company (Nanjing, China). MDA content was determined in accordance with the manufacturer's instructions.

### Detection of iNOS and COX-2 Levels

The colon was homogenated in pre-cold PBS to extract total protein. The homogenate was centrifuged at 15,000 g at 4 °C for 15 min. The levels of iNOS and COX-2 were measured by corresponding assay kits (Shanghai MLBIO Biotechnology Co. Ltd, China) according to the kit instructions.

### Western Blot Analysis

Colon tissues of all groups were homogenized using RIPA buffer [50 mM Tris (pH 7.4), 150 mM NaCl, 1% Triton X-100, 1% sodium deoxycholate, 0.1% SDS, 10 mM NaF, 5 mM EDTA, 1 mM Na_3_VO_4_] with protease inhibitor (1:100) on ice. The nuclear and cytoplasmic proteins were extracted using available nuclear and cytoplasmic protein extraction kits (Beyotime biotechnology, China) in accordance with the manufacturer's instructions. The concentration of protein was analyzed using the Bestbio BCA Protein Assay Kit (Shanghai, China). Protein lysates (30 μg) were separated by polyacrylamide gel-electrophoresis, and then transferred onto PVDF membranes (Millipore, Billerica, MA, USA). Whereafter, the membranes were blocked with 5% skim milk in TBST for 1 h and washed with TBST for three times. The membranes were incubated overnight at 4 °C with primary antibodies (Nrf2 or Keap1, diluted 1:2,000). The membranes were washed three times, and incubated with secondary antibodies (diluted 1:2,000) for 1 h. Histone H3 and β-actin served as the loading control, respectively. The chemiluminescence signals obtained were quantified with Image J software (National Institutes of Health, Bethesda, MD, USA).

### Real-Time PCR

Total RNA was extracted from the colon of all mice by TRIzol reagent (Thermo Scientific, MA, USA.) in accordance with the manufacturer's instruction. The primer sequences were presented in **Table 3**. RT-PCR was performed in a CFX96 Real-Time PCR Detection system (Bio-Rad, USA). The ampliﬁcation condition was as follows: a denaturation at 95°C for 30 s, and then 40 cycles at 95°C for 5 s and 60°C for 30 s. Relative mRNA expression levels of *GCLC*, *GCLM*, *HO-1* and *NQO1* were normalized with β-actin and calculated with the 2 ^−△△Ct^ method.

**Table 3:** Primer sequences.

Gene		Primer (5′ to 3′)
*GCLC*	Forward	TGGCTTTGAGTGCTGCATCT
	Reverse	ATCACTCCCCAGCGACAATC
*GCLM*	Forward	TCACAATGACCCGAAAGAACTG
	Reverse	ACCCAATCCTGGGCTTCAAT
*HO-1*	Forward	CAGCCCCACCAAGTTCAAAC
	Reverse	GGCGGTCTTAGCCTCTTCTGT
*NQO1*	Forward	GGTTTACAGCATTGGCCACACT
	Reverse	AACAGGCTGCTTGGAGCAAA
*ß-actin*	Forward	GGCTGTATTCCCCTCCATCG
	Reverse	CCAGTTGGTAACAATGCCATGT

### 16S rDNA Gene Sequencing and Gut Microbiota Analysis

The fecal samples in the colon of mice with ulcerative colitis were collected for the gut microbiota analysis (*n* = 8 for every group). Genomic DNA was extracted from every stool sample. USEARCH (v7.0.1090) was used to cluster tags of more than 97% identity into operational taxonomic units (OTUs), and the abundances of OTUs were calculated. Based on the OTUs and species annotation, the sample species complexity and species differences among groups were analyzed. OTU Venn diagram was performed by programming language R (v3.1.1) about OTU overlap in different groups. On the basis of OTU results, the diversity index and richness were further analyzed. The composition of flora structure can be analyzed at different levels of biological classification (from phylum to genus). Heatmap was analyzed to discover the specific microflora types.

### Statistical Analysis

Statistical analysis was performed by one-way analysis of variance (ANOVA) followed by Dunnett's test with SPSS 23.0 software (SPSS Inc., Chicago, IL, USA). Data were presented as the mean ± standard error of the mean (SEM). *p* < 0.05 was considered to indicate a statistically significant difference.

## Results

### BGF was Rich in Pinitol

**Figure 1:**
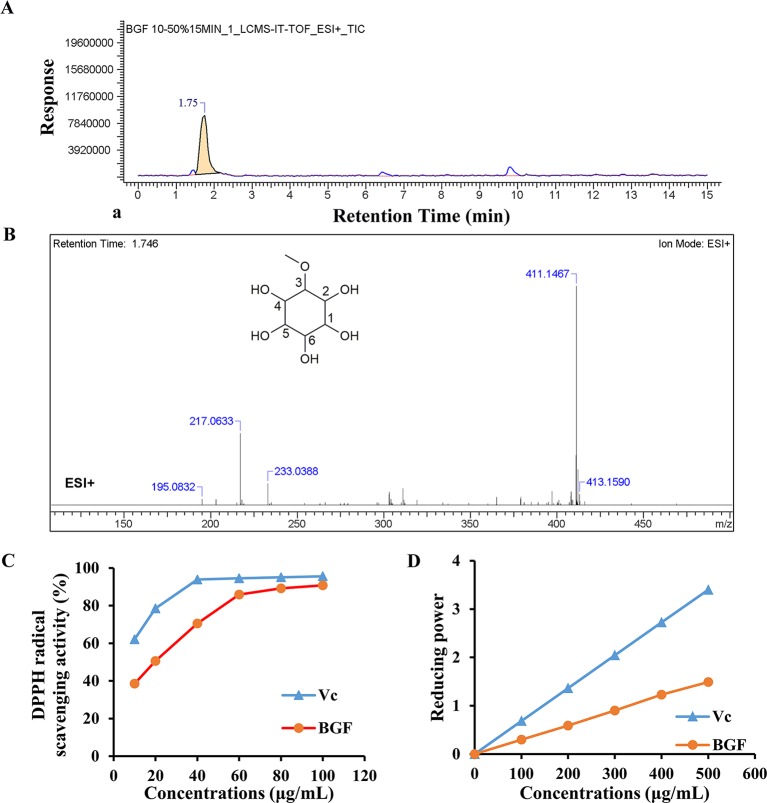
*Bruguiera gymnorrhiza fruit (BGF)* was rich in pinitol. **(A)** Ion chromatograms of BGF aqueous extract in positive mode. **(B)** MS spectra of BGF aqueous extract at the retention time of 1.746 min and chemical structure of pinitol. **(C)** DPPH scavenging activity of BGF aqueous extract. **(D)** Reducing power of BGF. Ascorbic acid (Vc) was used as a positive control.

LC-MS was used to identify the phytochemicals in lyophilized powder of BGF. The sample was analyzed in the positive and negative ion modes. In positive ion mode, compound 1 (C_7_H_14_O_6_) showed major fragments: [M + H]^+^ (*m/z* 195.0832), [M + Na]^+^ (*m/z* 217.0633), [M + K]^+^ (*m/z* 233.0388), [2M + Na]^+^ (*m/z* 411.1467), [2M + Na + 2H]^+^ (*m/z* 413.1590) (**Figure 1**). The ^13^C-NMR spectrum of BGF showed a characteristic signal of C-3 attached to OMe group appeared at δ: 82.35, 70.69 (C-1), 69.95 (C-2), 69.2 (C-4), 72.30 (C-5), 73.68 (C-6), and OMe appeared at δ: 57.52 (**Figure S2**). It was elucidated as pinitol by comparison with data reported previously (Ganbaatar et al., 2016; Tewari et al., 2017). High signals of pinitol in ^13^C-NMR revealed that lyophilized powder of BGF was rich in pinitol.

BGF and standard substance were analyzed, and the standard curve and linear range of pinitol were as follows: y = 4.9383x + 5.7598 (*r* ^2^ = 0.9990, 0.0354 ∼ 0.189 mg/mL). Based on the external standard method, the amounts of pinitol in BGF were calculated to be 402.3 mg/g aqueous extract.

### BGF Possessed Antioxidant Activity *in Vitro*

The DPPH radical scavenging ability of BGF was plotted in **Figure 1C**. DPPH radical scavenging ability was enhanced with increasing concentrations of BGF, which exhibited good DPPH scavenging activity when compared with the standard ascorbic acid. BGF possessed a strong scavenging activity of DPPH with the maximum suppression of 90.8% at the concentration of 100 μg/mL. The IC_50_ value was measured to be 20.45 μg/mL.

As presented in **Figure 1D**, the reducing activity of ascorbic acid and BGF was found to be directly proportional to the concentrations of the samples. The reducing power of BGF consistently increased with the elevating sample concentrations. At 500 mg/mL, the absorbance of reducing power was 1.49 for BGF.

### BGF Mitigated the Symptoms of DSS-Induced UC Mice

**Figure 2:**
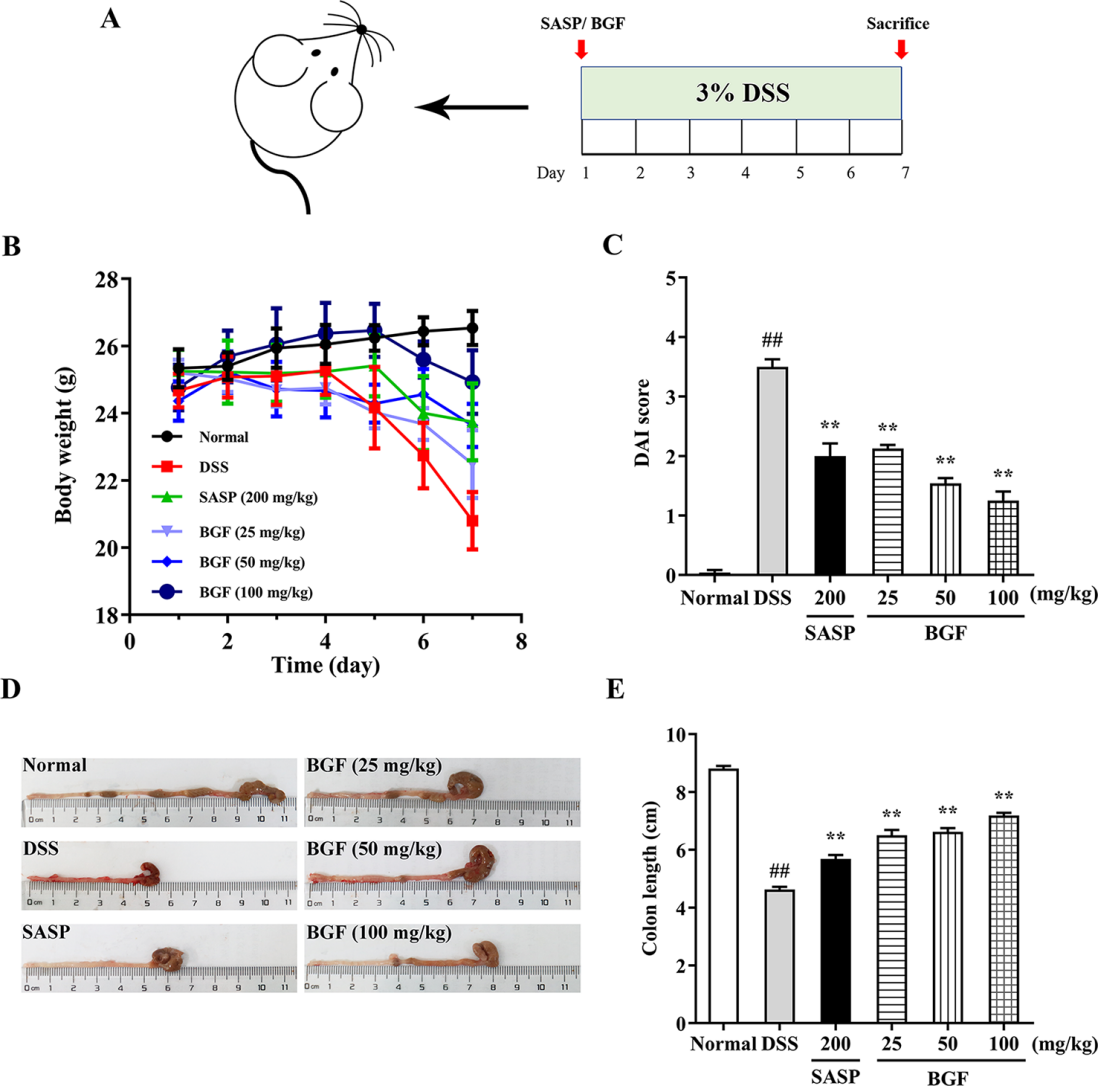
*Bruguiera gymnorrhiza fruit (BGF)* mitigated the symptoms of DSS-induced UC mice. **(A)** Experimental schedule. **(B)** Daily body weight. **(C)** Disease activity index (DAI). **(D)** Representative photographs of colon length. **(E)** Quantitative results of colon length among six groups. Data are expressed as mean ± SEM (*n* = 8). ^##^ *p* < 0.01 compared to normal group; **p < 0.01 compared to DSS group.

In the present study, 3% DSS in the drinking water induced various symptoms as observed in human UC. As shown in **Figure 2B**, a significant decrease in body weight was observed in DSS-treated mice as compared with that of the normal group, in which the body weight of mice was continuously rising. By contrast, the body weight loss induced by DSS was signiﬁcantly alleviated by treatment with SASP or BGF.

DAI score, an essential clinical manifestation of UC, is defined as an important indicator to evaluate the colonic injury (Samman et al., 2018). After administration with 3% DSS for 7 days, mice exhibited colitis-like symptoms, including diarrhea and bloody stool. The DAI score was significantly higher in the DSS group as compared with that of the normal group (**Figure 2C**, *p* < 0.01). However, treatment with BGF significantly decreased the DAI score in a dose-dependent manner (*p* < 0.01).

**Figure 2D** depicted the representative photographs of the colon in each group. The colons of mice in the DSS group displayed serious redness and bloody stools when compared with those of the normal group. However, the colons of BGF groups showed little redness or swelling. The colon length was inversely associated with the severity of colitis. To explore whether BGF exerted a beneficial effect on DSS-induced colonic shortening, the colon lengths of mice from different groups were comparatively measured. As shown in **Figure 2E**, the colon length dramatically decreased in DSS-induced mice as compared with that of the normal mice. However, the colons from mice in SASP and BGF groups were significantly longer than that of the DSS group. It is indicated that the experimental colitis induced by DSS was effectively inhibited by treatment with BGF.

### BGF Relieved the Colonic Injury and Inflammatory Infiltration in DSS-Induced UC Mice

**Figure 3:**
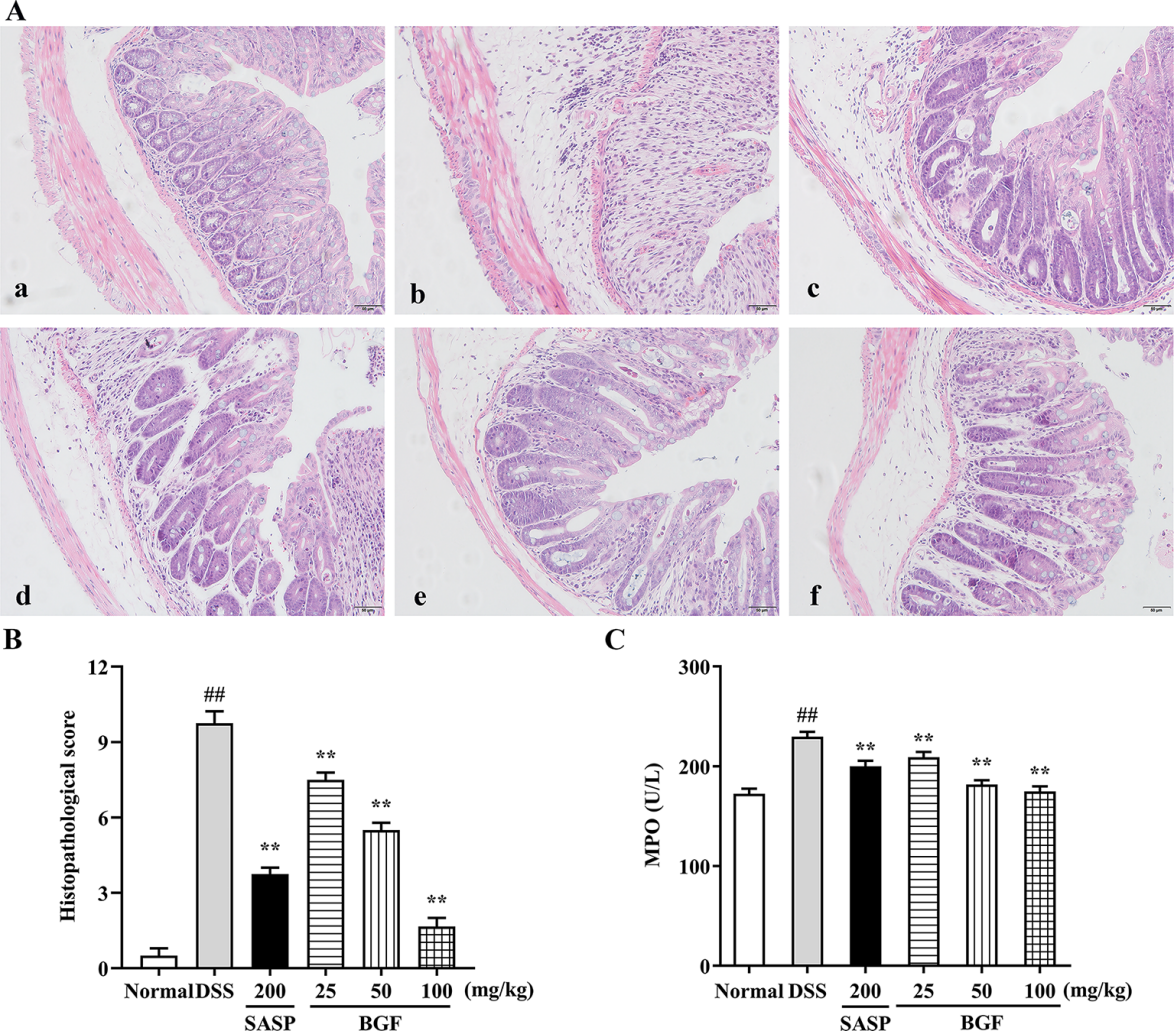
*Bruguiera gymnorrhiza fruit (BGF)* relieved the colonic injury in DSS-induced UC mice. **(A)** Representative photographs of H&E staining (magnification × 200). (a) Normal; (b) DSS; (c) SASP; (d) 25 mg/kg BGF; (e) 50 mg/kg BGF; (f) 100 mg/kg BGF. **(B)** Histopathological score. **(C)** MPO activity. Data are presented as mean ± SEM of eight mice in each group. ^##^ *p* < 0.01 compared to normal group; **p < 0.01 compared to DSS group.

Colonic histological changes were illustrated in the histological sections stained with hematoxylin and eosin. As depicted in **Figure 3A**, the colon tissues in control group were highly structured and the distribution between mucosa, submucosa, muscular layer, and outer membrane was clear and neatly arranged. DSS challenge caused noteworthy distortion of crypts and mucosa disruption of colons. On the contrary, BGF effectively improved the colon crypt structures and the surface epithelial injury. Oral administration with BGF (25, 50, and 100 mg/kg) resulted in prominent inhibition on the injuries and decreased the histopathological scores (**Figure 3B**, *p* < 0.01). In addition, high dose of BGF (100 mg/kg) exhibited superior effect to the positive drug SASP (200 mg/kg) in ameliorating these deteriorating histopathological alternations with smaller dosage. Our results indicate that treatment with BGF might display appreciably beneficial effect in attenuating DSS-induced colitis.

As shown in **Figure 3C**, MPO activity, a key index of infiltration of neutrophile granulocytes, significantly increased in DSS group. By contrast, there was a remarkable decrease in MPO activity of the BGF-treated mice as compared to that of the UC model mice (*p* < 0.01). These results suggest that BGF might mitigate the substantial grow of MPO-positive cells and neutrophils granulocyte infiltration in tissues.

### BGF Improved the Inflammatory Status in DSS-Induced UC Mice

**Figure 4:**
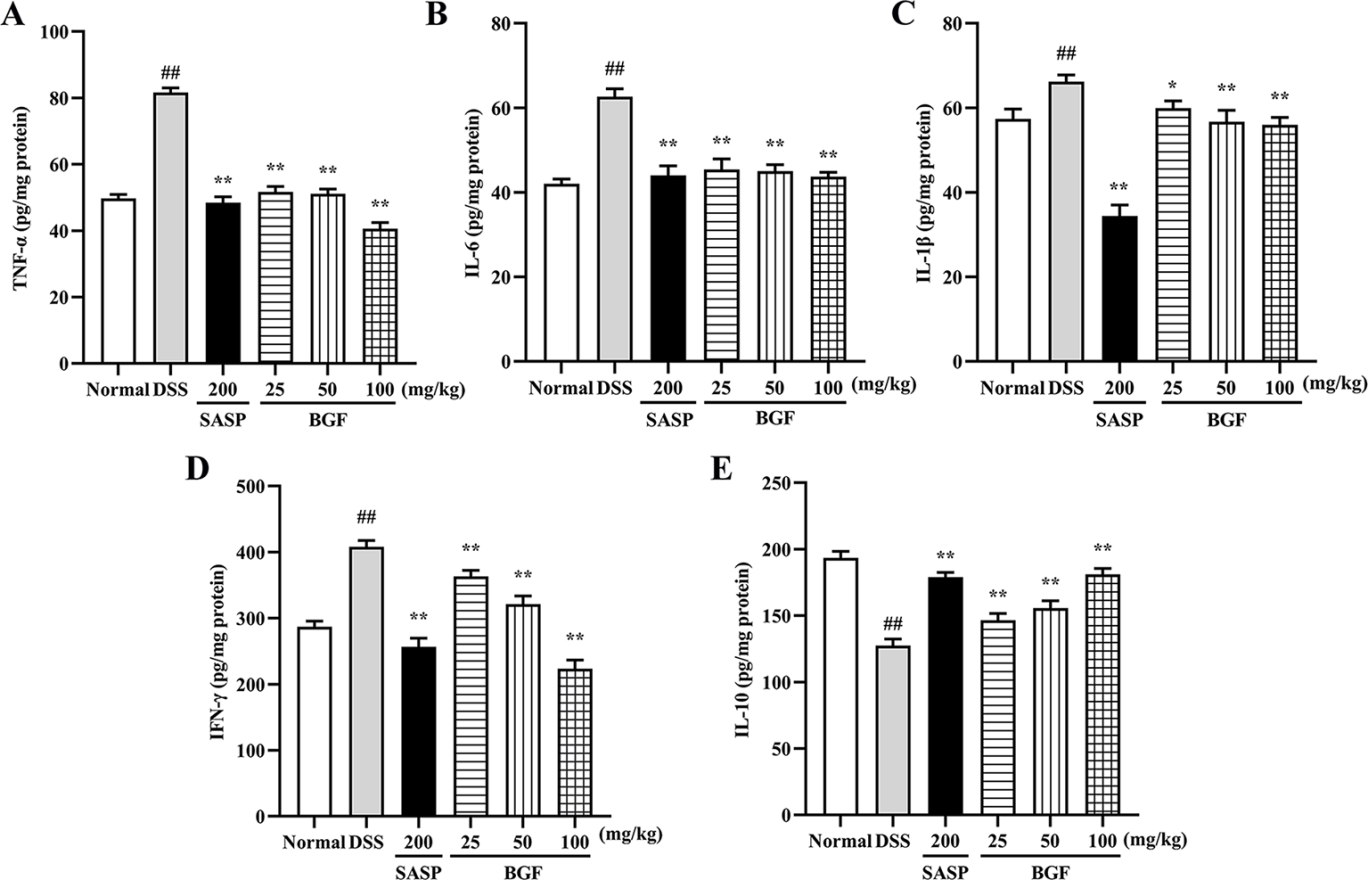
*Bruguiera gymnorrhiza fruit (BGF)* improved the inflammatory status in DSS-induced UC mice. The levels of inﬂammatory mediators: TNF-α **(A)**, IL-6 **(B)**, IL-1β **(C)**, IFN-γ **(D)** and IL-10 **(E)** in UC mice as measured by corresponding ELISA assay kits. Data are presented as mean ± SEM of eight mice in each group. ^##^ *p* < 0.01 compared to normal group; *p < 0.05, **p < 0.01 compared to DSS group.

To determine whether the protective effect of BGF against DSS-induced colitis was associated with cytokine productions, the colonic levels of TNF-α, IL-6, IL-1β, IFN-γ, and IL-10 were measured by corresponding ELISA kit. As shown in **Figure 4**, the levels of pro-inflammatory cytokines (TNF-α, IL-6, IL-1β, and IFN-γ) were markedly increased in mice of DSS model group (*p* < 0.01). Nevertheless, the accumulation of these cytokines was significantly decreased in BGF and SASP group (*p* < 0.01) as compared to that of the DSS group. On the other hand, BGF dramatically elevated the level of IL-10 in colitis mice in a dose-dependent manner compared to the DSS model group (*p* < 0.01).

### BGF Protected Colon From Oxidative Stress in DSS-Induced UC Mice

**Figure 5:**
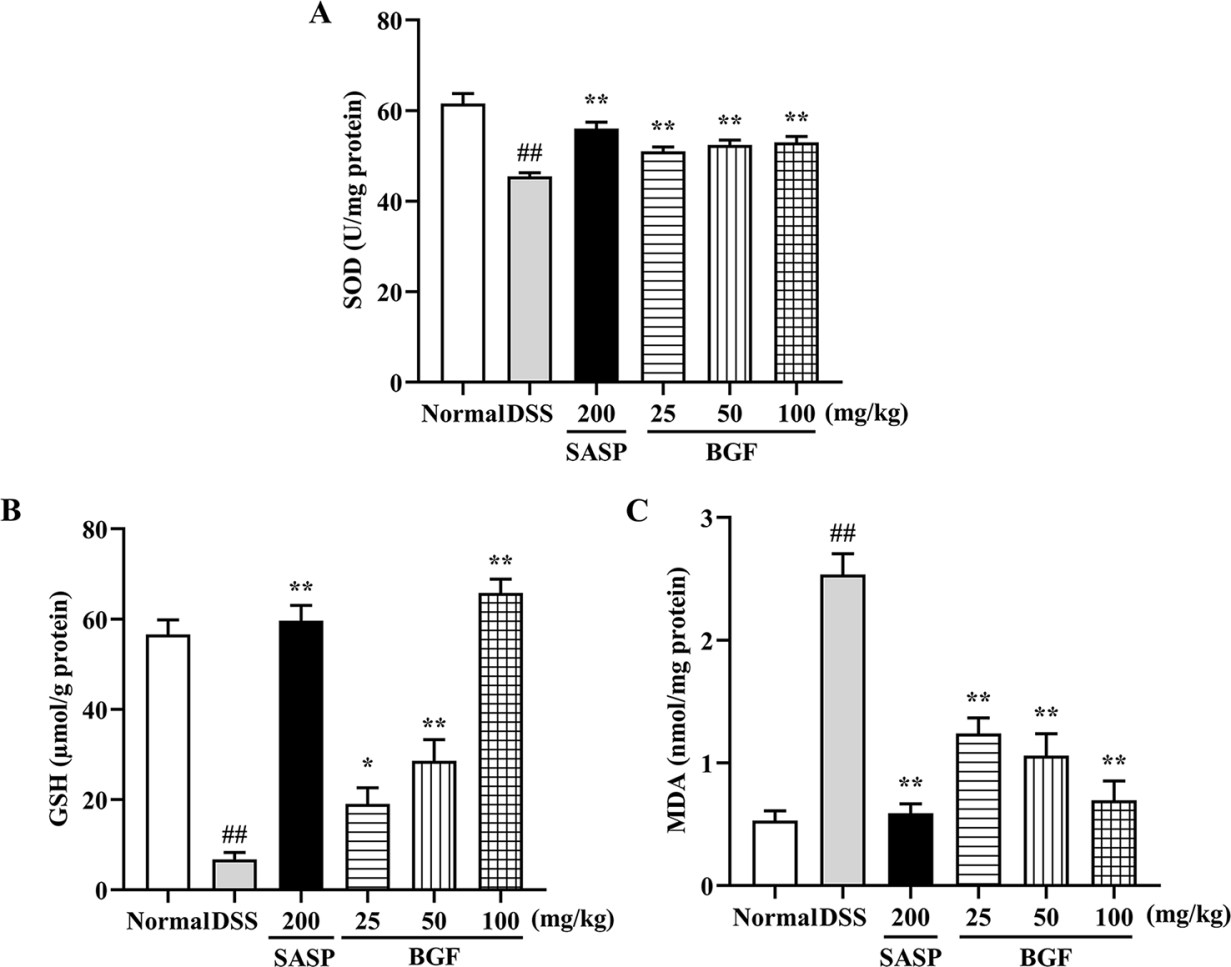
*Bruguiera gymnorrhiza fruit (BGF)* protected the colon from oxidative stress in DSS-induced UC mice. The levels of SOD **(A)**, GSH **(B)** and MDA **(C)**. Data are presented as mean ± SEM of eight mice in each group. ^##^ *p* < 0.01 compared to normal group; *p < 0.05, **p < 0.01 compared to DSS group.

As depicted in **Figure 5**, the levels of SOD and GSH of colitis mice were significantly decreased by 3% DSS compared with those of the normal mice (*p* < 0.01). When colitis mice were treated with BGF or SASP, the SOD and GSH levels were significantly increased in a dose-dependent manner. Additionally, as compared with that of the normal control group, the MDA content was significantly raised in colitis model group (*p* < 0.01). By contrast, the MDA level in colitis mice markedly decreased by treatment with BGF in a dose-dependent manner (*p* < 0.01).

### BGF Diminished the iNOS and COX-2 Levels

**Figure 6:**
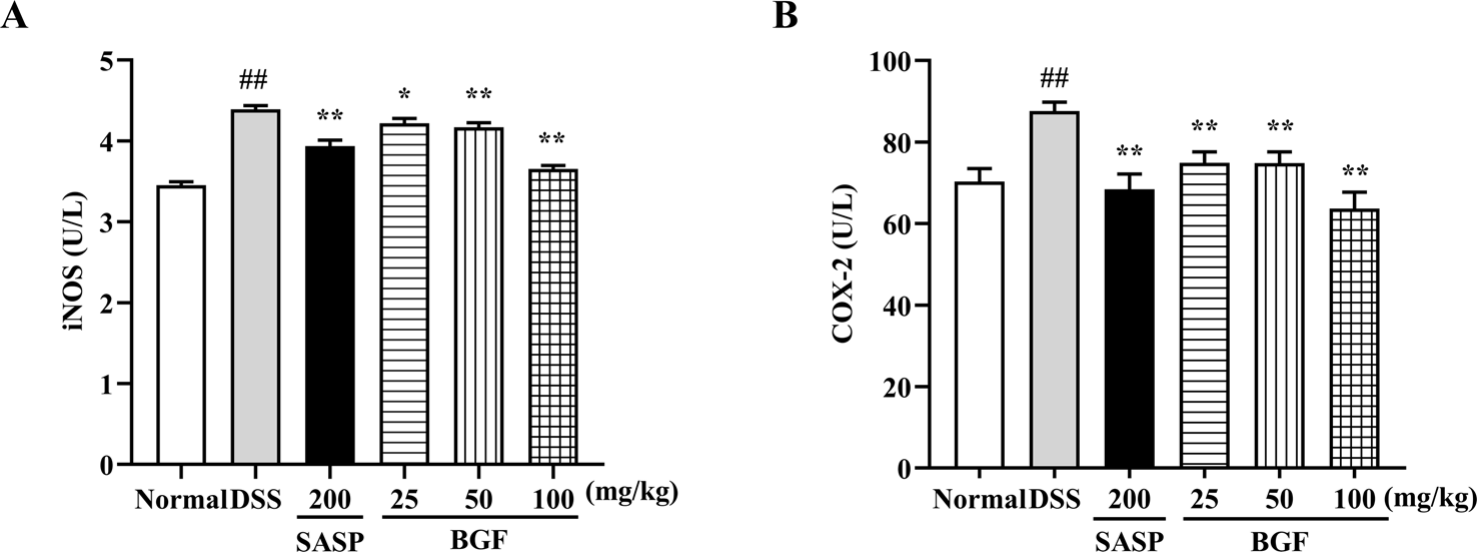
*Bruguiera gymnorrhiza fruit (BGF)* diminished the levels of iNOS and COX-2. The levels of iNOS **(A)** and COX-2 **(B)** as measured by ELISA. Data are presented as mean ± SEM of eight mice in each group. ^##^ *p* < 0.01 compared to normal group; *p < 0.05, **p < 0.01 compared to DSS group.

As presented in **Figure 6**, the DSS-induced colitis evidently promoted the levels of iNOS and COX-2 (*p* < 0.01) compared with the normal group. Nevertheless, treatment with SASP significantly suppressed the increased levels of iNOS and COX-2 induced by DSS (*p* < 0.01). Additionally, treatment with BGF exerted a remarkable inhibition on DSS-induced elevation of iNOS and COX-2 levels in a dose-dependent manner (*p* < 0.01).

### BGF Activated the Keap1/Nrf2 Signaling Pathway

**Figure 7:**
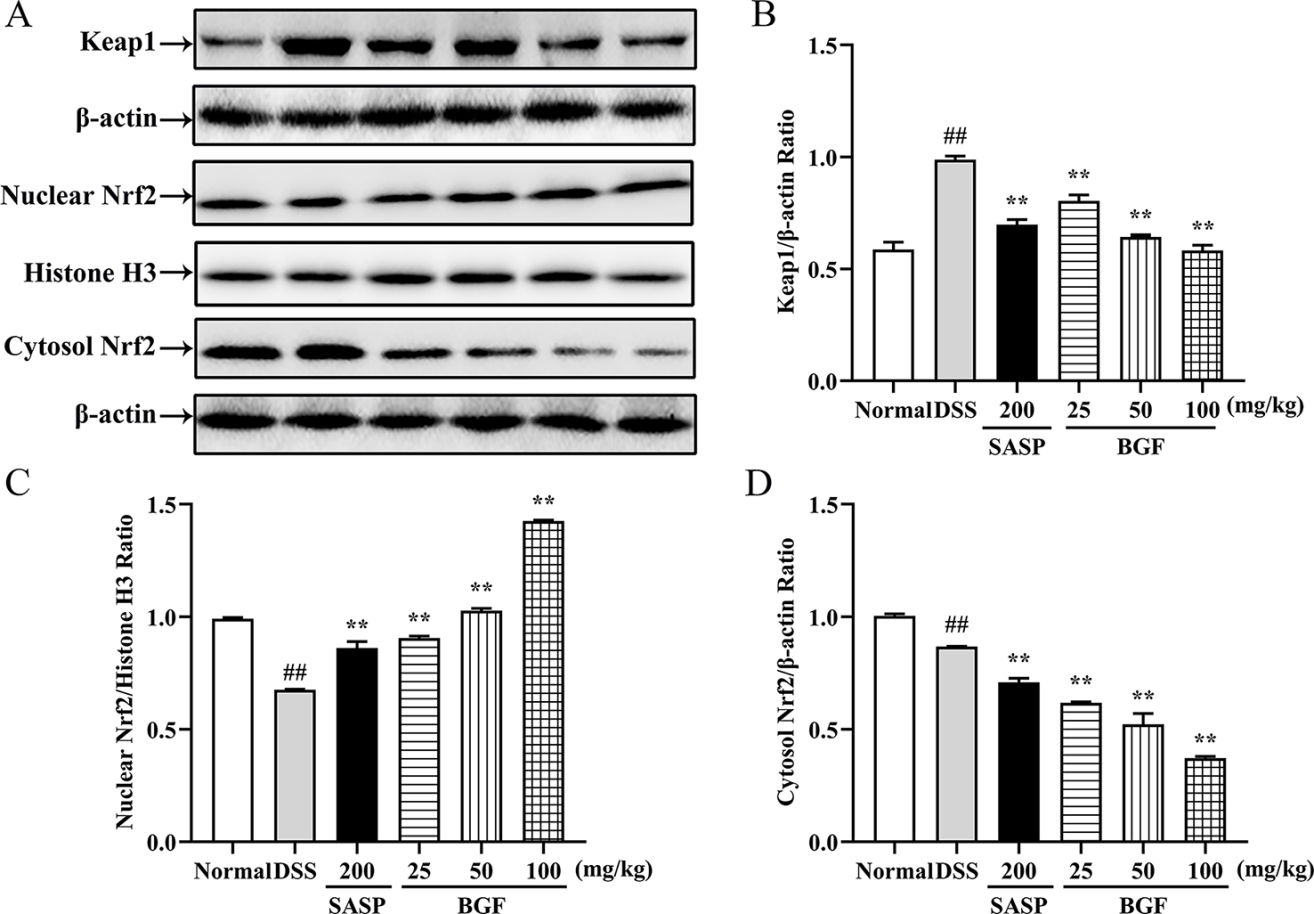
*Bruguiera gymnorrhiza fruit (BGF)* activated the Keap1/Nrf2 signaling pathway. **(A)** Representative expression bands of Keap1, nuclear Nrf2 and cytosolic Nrf2 by Western blot. Relative protein expression levels of Keap1 **(B)**, nuclear Nrf2 **(C)** and cytosolic Nrf2 **(D)** as detected by Western blot. Data are presented as mean ± SEM (*n* = 3). ^##^ *p* < 0.01 compared to normal group; **p < 0.01 compared to DSS group.

Keap1/Nrf2 signaling pathway, an important endogenous antioxidative stress pathway, plays an essential role in colon diseases (Bellezza et al., 2018). To assess whether BGF could mitigate the activation of Keap1/Nrf2 signaling pathway, we analyzed the protein expression levels of cytosolic Keap1, nuclear Nrf2 and cytosolic Nrf2 in the colon samples of mice by Western blot. As illustrated in **Figure 7**, the protein expression level of cytosolic Keap1 was signiﬁcantly elevated by DSS (p < 0.01) as compared with that of the normal mice. However, following BGF treatment, the cytosolic Keap1 protein level restored to almost the normal level (all p < 0.01). BGF exerted a greater inhibitory effect on cytosolic Keap1 protein expression and a superior effect on promoting the Nrf2 translocation as compared to SASP (p < 0.05). On another hand, down-regulated expression of cytosolic Nrf2 was observed in DSS group (p < 0.01) and BGF groups. The nuclear Nrf2 protein expression level was significantly reduced in DSS-induce mice (p < 0.01). Whereas the nuclear Nrf2 protein expression level in UC mice was markedly increased by treatment with BGF of three tested doses (25, 50, and 100 mg/kg, all *p* < 0.01).

### BGF Promoted the mRNA Expression of Nrf2 Downstream Genes

**Figure 8:**
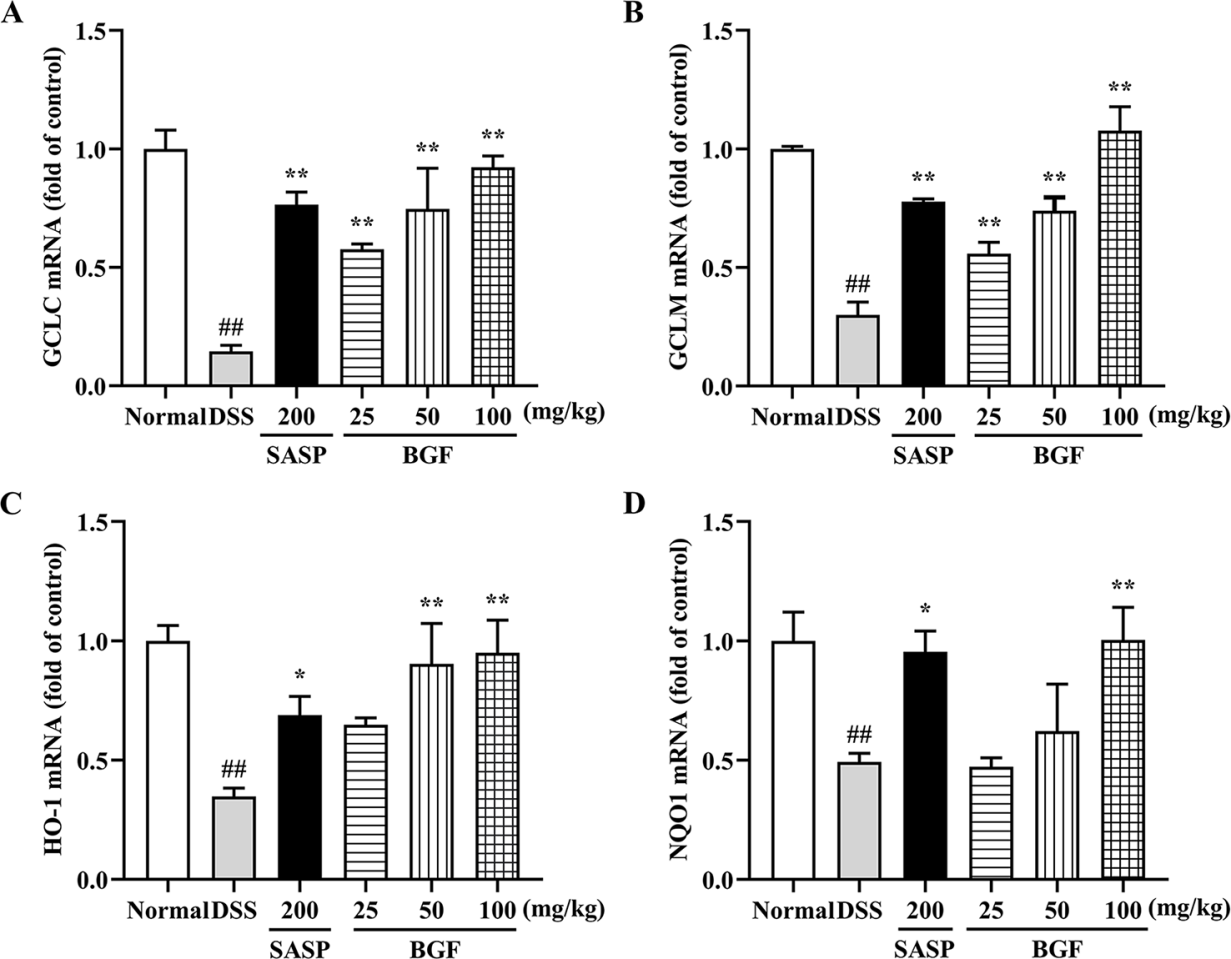
*Bruguiera gymnorrhiza fruit (BGF)* promoted the mRNA expression levels of Nrf2-downstream genes. Relative mRNA expression levels of GCLC **(A)**, GCLM **(B)**, HO-1 **(C)** and NQO1 **(D)** as measured by RT-PCR. Data are presented as mean ± SEM of six mice in each group. ^##^ *p* < 0.01 compared to normal group; *p < 0.05, **p < 0.01 compared to DSS group.

As shown in **Figure 8**, compared to the normal mice, there were remarkably lower expression levels of *GCLC*, *GCLM*, *HO-1* and *NQO1* in the model group (*p* < 0.01). Whereas there were significantly higher gene expression levels of *GCLC* (*p* < 0.01), *GCLM* (*p* < 0.01), *HO-1* and *NQO1* in mice treated with BGF as compared to those of the DSS group. Treatment with SASP also significantly up-regulated the mRNA levels of Nrf2 downstream genes.

### BGF Rebalanced Gut Microbiota in UC Mice

The relative abundance of each microorganism, including which microorganism was contained and the number of each microorganism sequence in the sample, could reveal the taxonomic comparison of one or more samples at each classiﬁcation level (**Figure S3**). In this study, Venn diagram and doughnut were adopted to intuitively and vividly represent the composition of microbial species at the genus level in each group of samples.

**Figure 9:**
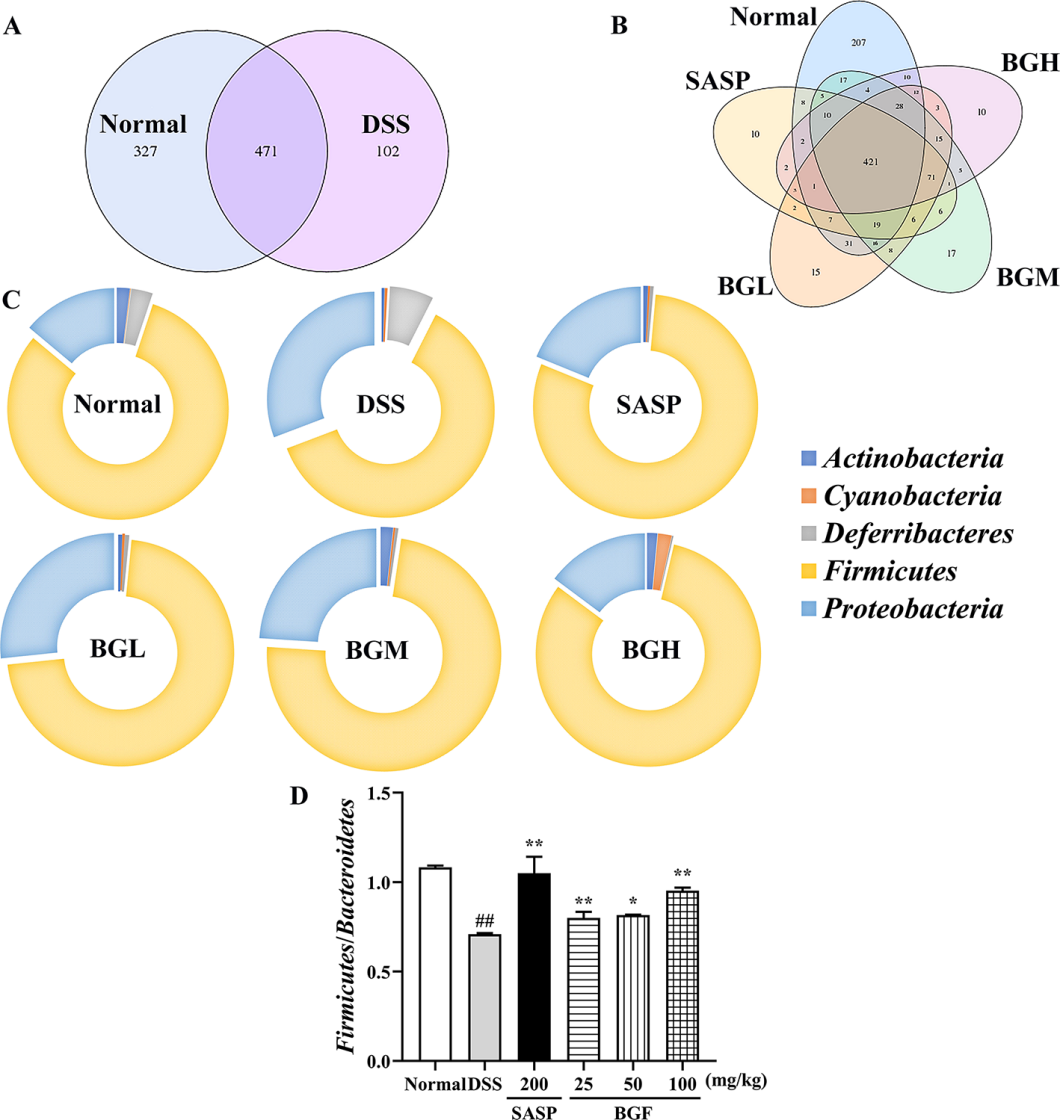
*Bruguiera gymnorrhiza fruit (BGF)* improved species abundance of intestinal flora in DSS-induced UC mice. **(A)** Venn diagram shows the number of OTU in normal and DSS groups. **(B)** Venn diagram shows the number of shared and individual OTU in normal, SASP, BGL (25 mg/kg BGF), BGM (50 mg/kg BGF), and BGH (100 mg/kg BGF) groups. **(C)** 16S rDNA gene sequencing analysis of the gut microbiota of mice at the phylum level. **(D)** The ratio of *Firmicutes*/*Bacteroidetes*. Data are presented as mean ± SEM of eight mice in each group. ^##^ *p* < 0.01 compared to normal group; *p < 0.05, **p < 0.01 compared to DSS group.

Venn diagram analyzed the number of OTU (operational taxonomic units), and the composition similarity and overlap relationship among different group samples were further comparatively investigated. As shown in **Figure 9A**, 207 OTUs were specific to the control group as compared with 10 OTUs in the DSS model group. Four hundred twenty-one OTUs were shared in the following four groups: 798 OTUs in normal group, 658 OTUs in BGF (25 mg/kg) group, 649 OTUs in BGF (50 mg/kg) group, and 598 OTUs in BGF (100 mg/kg) group, respectively (**Figure 9B**). These results indicated that the microbiota similarity of colitis mice evidently decreased compared with that of the normal group. However, treatment with BGF obviously improved the species composition in colitis mice, especially at the dose of 100 mg/kg.

In UC mice induced by DSS, results of phylum level analysis showed that *Bacteroidetes* and *Firmicutes* were dominant bacteria accounting for the largest proportion. The relative abundance of *Actinobacteria*, *Proteobacteria*, *Cyanobacteria*, and *Deferribacteres* was increased in the DSS group. However, the relative abundance of these organisms was significantly decreased after treatment with BGF. The relative abundance of *Bacteroidetes* obviously increased and that of *Firmicutes* decreased, which acted as the representative characters of the DSS-induced UC mice. The result was in accordance with the decreased ratio of *Firmicutes*/*Bacteroidetes* of UC mice in a previous report (Lee et al., 2018). After treatment with BGF, the ratio of *Firmicutes*/*Bacteroidetes* was elevated signiﬁcantly.

**Figure 10:**
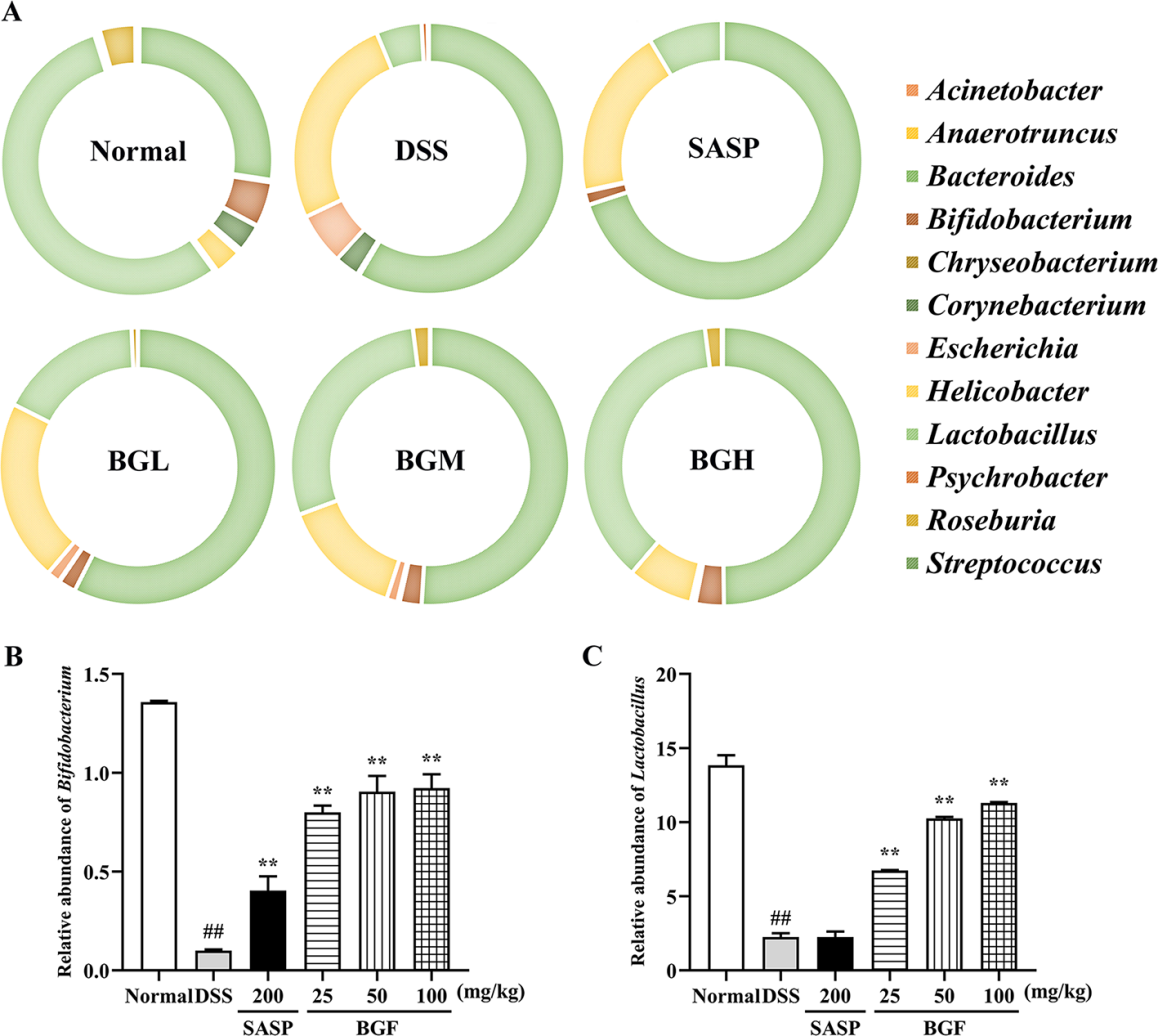
Effect of *Bruguiera gymnorrhiza fruit (BGF)* on the composition of gut microbiota at the genus level in normal, SASP, BGL (25 mg/kg BGF), BGM (50 mg/kg BGF) and BGH (100 mg/kg BGF) groups. **(A)** 16S rDNA gene sequencing analysis of the gut microbiota of mice at the genus level. Relative abundance of *Bifidobacterium* **(B)** and *Lactobacillus* **(C)**. Data are presented as mean ± SEM of eight mice in each group. ^##^ *p* < 0.01 compared to normal group; **p < 0.01 compared to DSS group.

The detailed information about the modulatory eﬀect of BGF on gut microbiota of UC rats was provided at genus level (**Figure 10A**). The results of genus level analysis showed that *Lactobacillus* and *Biﬁdobacterium* were beneﬁcial bacteria in the normal group, accounting for a large proportion. Compared to the normal group, levels of *Lactobacillus*, *Anaerotruncus*, and *Bifidobacterium* obviously declined in the DSS-induced colitis mice. The relative abundance of beneﬁcial bacteria in DSS group decreased obviously. In addition, the proportion of harmful bacteria (*Bacteroides* and *Helicobacter*) significantly increased following DSS treatment. By contrast, treatment with BGF effectively lowered the proportion of *Bacteroides* and *Streptococcus* (**Figure 10**). Furthermore, BGF noticeably facilitated the growth of probiotics, such as *Bifidobacterium*, *Anaerotruncus*, and *Lactobacillus*. The experimental results indicate that treatment with BGF could augment the number of beneficial bacteria and reduce the proportion of harmful bacteria to regulate the gut homeostasis.

## Discussion

*B. gymnorrhiza* (BG), a medicinal food plant, has been reported to display diverse biological properties (Ahmed et al., 2007). The fruit of BG (BGF) can serve as a vegetable in Solomon Island and is used as a food substitute for rice in a bad season (Allen and Duke, 2006). In China, Indonesia, and Fiji, BGF has been used as a traditional therapy against diarrheal (Nopara, 1999) and diabetes for years. However, the endeavor to illuminate its important traditional applications like dysentery is still rare, and current exploration of BGF on colitis is inadequate. Based on the ethnopharmacological application of BG, the present study investigated the protective effect of BGF on DSS-induced colitis and expounded its potential mechanism.

The main component potentially contributing to the bioactivity of BGF, was identified and quantitated via LC-MS, NMR (**Figure S1** and **S2**) and HPLC. Pinitol was found to be the major component of aqueous extract of BGF, with the concentration of 402.3 mg/g aqueous extract. Pinitol has been reported to harbor appreciable antioxidative, antidiarrheal, antitumor, and antidiabetic activities (Alonso-Castro et al., 2018). In order to investigate the potential antioxidant activity of BGF, DPPH radical scavenging ability and reducing power assay were performed. DPPH assay was generally used to evaluate the free radical scavenging capacity of natural antioxidant (Shi et al., 2016). In the present study, BGF exerted powerful antioxidative effect *in vitro* (with IC_50_ of 20.45 μg/mL for DPPH assay). And the absorbance of reducing power was 1.49 for BGF at 500 mg/mL. Considering the fact that antioxidant strategy serves an important role in treating UC, and pinitol possesses antioxidative and antidiarrheal effects, we hypothesized that the therapeutic effect of BGF observed may be closed associated with the presence of pinitol. Previous report has shown the presence of other phytochemical constituents like phenolic compounds, terpenoids, glycosides (Roy et al., 2018) and tannins (Lin et al., 2006) in the aqueous extract of BGF. It has been reported that tannins exert antioxidative and anti-diarrhea effects (Russo et al., 2018). It is possible to hypothesize that the modulatory effect of BGF is mediated by the pinitol, other active compounds, or their synergistic effects.

In the present work, the colitis model induced by oral administration with 3% DSS, manifested symptoms similar to patients with colitis, such as diarrhea, hemafecia, weight loss, and colon length shortening, which were in accord with preceding findings (Sha et al., 2013). DAI score usually represents the severity of DSS-induced colitis. Colon length is regarded as an indirect index inversely associated with the severity of colitis (Huang et al., 2019). In the present study, the DSS-induced mice exhibited higher DAI score, histopathological score, lower colon length as compared with the normal mice. However, treatment with BGF not only significantly decreased the DAI and regained the colon length, but also dose-dependently lowered the histopathological score by mitigating the mucosa epithelial injury. The anti-colitis effect of BGF was even superior to that of the positive control SASP with much smaller dosage. Neutrophils infiltration into the inflamed mucosa is one of the most distinct histological characteristics in the inflamed colonic mucosa of UC. Treatment with BGF obviously attenuated colonic histopathological deterioration including mucosa injury, loss of crypts, inflammatory infiltration of submucosa in colon when compared with DSS model group. MPO activity is a major factor reflecting granulocyte infiltration into colonic tissues (Deroche et al., 2014). In the current work, BGF was observed to conspicuously decrease the colonic MPO level elicited by DSS in a dose-dependent manner. BGF seemed to exert superior protective effect to SASP against granulocyte infiltration of UC. The results obtained collectively indicate that treatment with BGF effectively attenuate the established colonic inflammation and exhibit a pronouncedly protective effect against DSS-induced colitis in mice.

UC is a relapsing inflammatory disease featured by dysregulated immune response and disequilibrium of cytokine release. In the pathogenesis of UC, the intestinal mucosal immune system is out of balance, which is manifested by the imbalance of levels of pro-inflammatory cytokines (e.g. TNF-α, IL-6, IL-1β, and IFN-γ) and anti-inflammatory cytokines (e.g. IL-10). Therefore, favorable regulation of these inflammatory mediators could provide a feasible therapeutic strategy for the treatment of UC. A previous study has shown that protocatechuic acid could adjust inflammatory cytokines as well as inhibited COX-2 and iNOS to alleviate UC (Farombi et al., 2016). In this study, treatment with BGF effectively inhibited the uncontrolled release of TNF-α, IL-6, IL-1β, and IFN-γ, and increased the level of IL-10 as compared with the UC mice (all *p* < 0.01). COX-2 and iNOS, known as inducible enzymes rapidly expressed by mononuclear macrophages, fibroblast or other cells after stimulated within the body, are tightly associated with the pathogenesis of UC. When iNOS and COX-2 are excessively released, oxidative injury can occur (Bellezza et al., 2018; Mo et al., 2018). In this study, the elevated levels of iNOS and COX-2 induced by DSS were noticeably suppressed by treatment with BGF in a dose-dependent manner (**Figure 6**). The results suggest that the ameliorative effect of BGF against DSS-induced colitis may be closely associated with its favorable regulation of inflammatory cytokine productions, and iNOS and COX-2 levels.

Oxidative stress is one of the most common pathogenic factors leading to inflammatory diseases. UC is a peroxidative colon disease, and there are many intestinal pathological changes associated with the process of colitis (Kuwahara et al., 2017). Oxidative stress is a response of the body stimulated by DSS. It may suppress the endogenous defense systems that regulate ROS production (Bhattacharyya et al., 2014). High levels of ROS have been reported to result in oxidative stress and DNA damage due to an imbalance between innate and exogenous antioxidants and ROS (Rezaie et al., 2007). Oxidative stress indicators (SOD, MDA, and GSH) belong to the phase II control detoxifying enzymes of Nrf2. In this study, treatment with BGF significantly reduced the accumulation of MDA and promoted the levels of SOD and GSH in colonic tissues (**Figure 5**, all *p* < 0.01). The results indicated that BGF may enhance the antioxidative system and suppress the lipid peroxidation, which is postulated to contribute to relieving DSS-induced UC in mice.

Keap1/Nrf2 signaling pathway, an important endogenous antioxidant pathway, serves an essential role in UC (Lu et al., 2016). Nrf2 is an important transcription factor regulating cellular antioxidative stress. In normal physiological conditions, cytoplasmic protein Keap1 binds with Nrf2. Under oxidative stress, Nrf2 decouples with Keap1 into nuclear (Li et al., 2013). After reacting with antioxidant response element (ARE), Nrf2 initiates the phase II control detoxifying enzymes and antioxidant enzyme genes (*GCLC*, *GCLM*, *HO-1* and *NQO1*), increases cell resistance against oxidative stress to exert a protective effect. Disruption or loss of Keap1/Nrf2 signaling resulted in enhanced susceptibility not only to oxidative stress but also to inflammatory tissue injuries (Kim et al., 2010). In the present study, compared to the DSS group, the nuclear Nrf2 expression was significantly increased in BGF and SASP treatment groups (*p* < 0.01). While the Keap1 expression level was dose-dependently downregulated in BGF-treated mice (*p* < 0.01). Therefore, inhibition of Keap1 may active the Keap1/Nrf2 pathway against oxidative stress. When Keap1/Nrf2 pathway is activated, downstream genes controlled by Nrf2 including *GCLC*, *GCLM*, *HO-1* and *NQO1* are also activated (Sekhar et al., 2003). Following treatment with BGF, the mRNA expression of *GCLC*, *GCLM*, *HO-1* and *NQO1* significantly increased in a dose-dependent manner. Therefore, BGF is supposed to restore the redox balance, at least partially, through the activation of Keap1/Nrf2 signaling pathway, thereby suppressing the oxidative stress and inflammatory response and impeding the exacerbation of colitis injury.

The gastrointestinal tract of mammals is a house for microbial community, collectively known as the intestinal flora. Intestinal microbiota plays a momentous impact on the development of DSS-induced UC (Glymenaki et al., 2017). The severity of gut microbiota disorder directly affects the rate of inflammation progression (Zhang et al., 2018). It has been reported that microbiota composition change of colon cancer could affect mucosal immune response (Sobhani et al., 2011). Indeed, treatment of DSS-induced acute colitis model with prebiotics and probiotics has been reported to attenuate the symptoms of disease and prevent inflammation (Zhou et al., 2019). In this study, high-throughput sequencing was used to analyze the data in bioinformatics. Venn diagram showed that treatment with BGF recovered the bacterial diversity of the gut and effectively improved the species composition in DSS-induced mice (**Figure 9**). Therefore, BGF may favorably restore the microbiota abundance by regulating the bacteria compositional changes that are associated with UC.

Gut microbiota is a key determinant of the onset and severity of DSS-induced colitis. Under normal circumstances, probiotics account for the majority of intestinal flora. When intestinal flora is out of balance, harmful microbiota grows excessively, which contributes to the occurrence of colon disease (Nagalingam et al., 2011). It has been indicated that prevention of oxidative stress injury by specific groups of commensal obligate anaerobic bacteria may exert protective effects against pathogens. In the present work, there was a signiﬁcant diﬀerence in phylum and genus classiﬁcation of the gut microbiota between the DSS and BGF groups, indicating that BGF can improve the dysbacteriosis of microbial diversity and population in the mouse colon. The ratio of *Firmicutes*/*Bacteroidetes*, an important indicator of intestinal ﬂora structure change, can be used to indicate UC (Kataoka, 2016). In the present work, treatment with BGF was observed to significantly promote *Firmicutes* and inhibit *Bacteroidetes*, resulting in enhanced *Firmicutes*/*Bacteroidetes* ratio which was decreased by DSS. At the genus level, *Streptococcus* has been reported to increasingly present in patients with colorectal cancer (CRC) (Kuda et al., 2017). In the present work, *Streptococcus* was observed to be relatively increased in UC mice. Additionally, it has been reported that *Bacteroides* could induce the emission of regulatory T cells and reduce intestinal injury (Atarashi et al., 2013). In the present study, the pathogenic bacteria (*Bacteroides*) in DSS-induced colitis mice was significantly enriched, which is congruent with previous investigation (Wu et al., 2018). Compared with the microbiota of healthy individuals, patients with IBD has a reduced number of protective bacteria (*Bifidobacterium* and *Lactobacillus*), which has immunomodulatory effects (Coqueiro et al., 2019). Our result indicated that the proportion of probiotics (*Bifidobacterium*, *Anaerotruncus*, and *Lactobacillus*) content obviously decreased in DSS-induced colitis mice, which are in line with previous reports (Dong et al., 2012). By contrast, treatment with BGF enhanced the relative abundance of probiotics, including *Bifidobacterium* and *Anaerotruncus*, and inhibited the colonization of pathogenic bacteria (*Bacteroides*, *Corynebacterium*, and *Streptococcus*) as compared to the DSS model group. *Lactobacillus*, a probiotic, exerted beneficial eﬀects on inflammatory bowel disorders by stimulating immune cells (Liu et al., 2018). Specifically, the relative abundance of *Lactobacillus* was found to be increased in BGF group, indicating that BGF could protect intestinal tract by increasing the probiotics (*p* < 0.01). Colitis is regarded as an immune-mediated disorder resulting from the abnormal interaction between intestinal microbes and the local immune system (Gao et al., 2018). Some probiotics may naturally exhibit anti-inflammatory effects when interacting with the human gut microbiota (Lee and Kim, 2019). And microbial changes occur while the inflammatory and oxidative status was stimulated by DSS. The pathological characteristics of UC suggest a link between these seemingly disparate mechanisms. Taken together, treatment with BGF could promote the microbial diversity and the growth of probiotics in the intestinal tract, and inhibit the colonization of pathogenic bacteria, which contributes to the maintenance of intestinal homeostasis and the potential improvement of the host's inflammatory and oxidative status.

To the best of our knowledge, this investigation is the first effort to demonstrate the protective effect of BGF against murine experimental UC, and delineate its potential underlying mechanism. The results presented herein corroborate the folk medicinal use of *B. gymnorrhiza* fruit in the treatment of diarrhea, and indicate the potential of BGF to be further developed into a promising food-derived candidate for the treatment of colitis.

## Conclusion

BGF possesses pronounced protective effect against DSS-induced murine experimental colitis, which is even superior to the positive drug SASP with smaller dosage. The protective mechanism of BGF may involve the attenuation of inflammation, activation of Keap1/Nrf2 signaling pathway, and favorable regulation of gut microbiota. This study provides experimental evidence to support the traditional application of *B. gymnorrhiza* fruit in the treatment of dysentery. The result indicates that BGF has potential to be further developed into a promising therapeutic candidate against ulcerative colitis.

## Data Availability Statement

The datasets generated for this study can be found in NCBI using the accession number PRJNA593171.

## Ethics Statement

The mice were obtained from Guangzhou University of Chinese Medicine. This study was performed based on the National Institutes of Health guide for the care and use of laboratory animals (NIH Publications No. 8023). The experimental protocols followed the Animal Ethics Committee of Guangzhou University of Chinese Medicine.

## Author Contributions

All authors including YL, XZ, JCh, DL, JX, ZiS, XH, XY, LW, JCa and ZhS have made substantial, direct and intellectual contribution to the work and approved it for publication.

## Funding

This work was supported by grants from the National Key R&D Program of China (No.2017YFC0506200), the Guangdong Forestry Science and Technology Innovation Program (No.2016KJCX026), the Science and Technology Development Special Project of Guangdong Province (No.2017A050506044), the Guangdong Provincial Department of Education Feature Innovation Project (No.2016KTSCX018), and the Science and Technology Project of Guangzhou (No.201704030028).

## Conflict of Interest

The authors declare that the research was conducted in the absence of any commercial or financial relationships that could be construed as a potential conflict of interest.

## Abbreviations

ANOVA, Analysis of variance; COX-2, Cyclooxygenase-2; DAI, Disease activity index; DSS, Dextran sulfate sodium; ELISA, Enzyme-linked immunosorbent assay; GCLC, Glutamate-cysteine ligase catalytic subunit; GCLM, Glutamate-cysteine ligase modifier subunit; GSH, Glutathione; HO-1, Heme oxygenase-1;IBD, Inflammatory bowel disease; IFN-γ, Interferon-γ; IL-1β, Interleukin-1β; IL-10, Interleukin-10; IL-6, Interleukin-6; iNOS, Inducible nitric oxide Synthase; MDA, Malondialdehyde; SASP, Salicylazosulfapyridine; MPO, Myeloperoxtidase; NQO1, NAD(P)H quinone dehydrogenase 1; Nrf2, NF-E2-related factor 2; OTU, Operational taxonomic units; SOD, Superoxide dismutase; TBA, Thiobarbituric acid; TNF-α, Tumor necrosis factor-α; UC, Ulcerative colitis. Vc, Ascorbic acid.

## References

[B1] Ahmed F. Shahid I. Gain N. Reza M. Sadhu S. J. O. P. Medicine E. (2007). Antinociceptive and antidiarrhoeal activities of *Bruguiera gymnorrhiza*. Orient. Pharm. Exp. Med. 7 (3), 280–285. 10.3742/OPEM.2007.7

[B2] Allen J. A. Duke N. C. J. E. (2006). *Bruguiera gymnorrhiza* (large-leafed mangrove). Permanent Agric. Resour.

[B3] Alonso-Castro A. J. Zapata-Morales J. R. Arana-Argaez V. Torres-Romero J. C. Ramirez-Villanueva E. Perez-Medina S. E. (2018). Pharmacological and toxicological study of a chemical-standardized ethanol extract of the branches and leaves from Eysenhardtia polystachya (Ortega) Sarg. (Fabaceae). J. Ethnopharmacol 224, 314–322. 10.1016/j.jep.2018.06.016 29913299

[B4] Asto E. Mendez I. Audivert S. Farran-Codina A. Espadaler J. (2019). The efficacy of probiotics, prebiotic inulin-type fructans, and synbiotics in human ulcerative colitis: a systematic review and meta-analysis. Nutrients 11 (2), 293. 10.3390/nu11020293 PMC6412539 30704039

[B5] Atarashi K. Tanoue T. Oshima K. Suda W. Nagano Y. Nishikawa H. (2013). Treg induction by a rationally selected mixture of Clostridia strains from the human microbiota. Nature 500 (7461), 232–236. 10.1038/nature12331 23842501

[B6] Bandaranayake W. M. J. M. Marshes S. (1998). Traditional and medicinal uses of mangroves. Mangroves Salt Marshes 2 (3), 133–148. 10.1023/a:1009988607044

[B7] Bellezza I. Giambanco I. Minelli A. Donato R. (2018). Nrf2-Keap1 signaling in oxidative and reductive stress. Biochim. Biophys. Acta Mol. Cell Res. 1865 (5), 721–733. 10.1016/j.bbamcr.2018.02.010 29499228

[B8] Bhattacharyya A. Chattopadhyay R. Mitra S. Crowe S. E. (2014). Oxidative stress: an essential factor in the pathogenesis of gastrointestinal mucosal diseases. Physiol. Rev. 94 (2), 329–354. 10.1152/physrev.00040.2012 24692350 PMC4044300

[B9] Coqueiro A. Y. Raizel R. Bonvini A. Tirapegui J. Rogero M. M. (2019). Probiotics for inflammatory bowel diseases: a promising adjuvant treatment. Int. J. Food Sci. Nutr. 70 (1), 20–29. 10.1080/09637486.2018.1477123 29804478

[B10] Deroche T. C. Xiao S. Y. Liu X. J. G. R. (2014). Histological evaluation in ulcerative colitis. Gastroenterol. Rep. 2 (3), 178–192. 10.1093/gastro/gou031 PMC4124271 24942757

[B11] Dong L. Li J. Liu Y. Yue W. Luo X. (2012). Toll-like receptor 2 monoclonal antibody or/and Toll-like receptor 4 monoclonal antibody increase counts of Lactobacilli and Bifidobacteria in dextran sulfate sodium-induced colitis in mice. J. Gastroenterol. Hepatol 27 (1), 110–119. 10.1111/j.1440-1746.2011.06839.x 21722182

[B12] Eisenstein M. (2018). Ulcerative colitis: towards remission. Nature 563 (7730), S33. 10.1038/d41586-018-07276-2 30405234

[B13] Erben U. Loddenkemper C. Doerfel K. Spieckermann S. Haller D. Heimesaat M. M. (2014). A guide to histomorphological evaluation of intestinal inflammation in mouse models. Int. J. Clin. Exp. Pathol. 7 (8), 4557–4576. 25197329 PMC4152019

[B14] Farombi E. O. Adedara I. A. Awoyemi O. V. Njoku C. R. Micah G. O. Esogwa C. U. (2016). Dietary protocatechuic acid ameliorates dextran sulphate sodium-induced ulcerative colitis and hepatotoxicity in rats. Food Funct. 7 (2), 913–921. 10.1039/c5fo01228g 26691887

[B15] Ganbaatar C. Gruner M. Tunsag J. Batsuren D. Ganpurev B. Chuluunnyam L. (2016). Chemical constituents isolated from Zygophyllum melongena Bunge growing in Mongolia. Nat. Prod Res. 30 (14), 1661–1664. 10.1080/14786419.2015.1118630 26795069

[B16] Gao X. Cao Q. Cheng Y. Zhao D. Wang Z. Yang H. (2018). Chronic stress promotes colitis by disturbing the gut microbiota and triggering immune system response. Proc. Natl. Acad. Sci. 115 (13), E2960–E2969. 10.1073/pnas.1720696115 29531080 PMC5879702

[B17] Glymenaki M. Singh G. Brass A. Warhurst G. McBain A. J. Else K. J. (2017). Compositional changes in the gut mucus microbiota precede the onset of colitis-induced inflammation. Inflammatory bowel Dis. 23 (6), 912–922. 10.1097/MIB.0000000000001118 28498157

[B18] He Y. Yu H. Y. Ge Y. X. Li X. T. Jiang M. S. Liu Y. L. (2019). Bacterial beta-glucuronidase alleviates dextran sulfate sodium-induced colitis in mice: a possible crucial new diagnostic and therapeutic target for inflammatory bowel disease. Biochem. Biophys. Res. Commun. 513 (2), 426–433. 10.1016/j.bbrc.2019.03.196 30967260

[B19] Huang Y. F. Li Q. P. Dou Y. X. Wang T. T. Qu C. Liang J. L. (2019). Therapeutic effect of Brucea javanica oil emulsion on experimental Crohn's disease in rats: involvement of TLR4/NF-kappaB signaling pathway. BioMed. Pharmacother. 114, 108766. 10.1016/j.biopha.2019.108766 30901719

[B20] Kaplan G. G. (2015). The global burden of IBD: from 2015 to 2025. Nat. Rev. Gastroenterol. Hepatol 12 (12), 720–727. 10.1038/nrgastro.2015.150 26323879

[B21] Kataoka K. (2016). The intestinal microbiota and its role in human health and disease. J. Med. investigation: JMI 63 (1-2), 27–37. 10.2152/jmi.63.27 27040049

[B22] Kim J. Cha Y. N. Surh Y. J. (2010). A protective role of nuclear factor-erythroid 2-related factor-2 (Nrf2) in inflammatory disorders. Mutat. Res. 690 (1-2), 12–23. 10.1016/j.mrfmmm.2009.09.007 19799917

[B23] Kitakaze T. Makiyama A. Samukawa Y. Jiang S. Yamashita Y. Ashida H. (2019). A physiological concentration of luteolin induces phase II drug-metabolizing enzymes through the ERK1/2 signaling pathway in HepG2 cells. Arch. Biochem. Biophys. 663, 151–159. 10.1016/j.abb.2019.01.012 30641047

[B24] Kuda T. Yokota Y. Shikano A. Takei M. Takahashi H. Kimura B. (2017). Dietary and lifestyle disease indices and caecal microbiota in high fat diet, dietary fibre free diet, or DSS induced IBD models in ICR mice. J. Funct. Foods 35, 605–614. 10.1016/j.jff.2017.06.030

[B25] Kuwahara E. Murakami Y. Nakamura T. Inoue N. Nagahori M. Matsui T. (2017). Factors associated with exacerbation of newly diagnosed mild ulcerative colitis based on a nationwide registry in Japan. J. Gastroenterol. 52 (2), 185–193. 10.1007/s00535-016-1209-x 27075755

[B26] Lee C. S. Kim S. H. (2019). Anti-inflammatory and anti-osteoporotic potential of lactobacillus plantarum A41 and L. fermentum SRK414 as probiotics. Probiotics Antimicrobial Proteins (1), 1–12. 10.1007/s12602-019-09577-y 31372901

[B27] Lee W. T. Tung Y. T. Wu C. C. Tu P. S. Yen G. C. (2018). Camellia oil (Camellia oleifera Abel.) Modifies the Composition of Gut Microbiota and Alleviates Acetic Acid-Induced Colitis in Rats. J. Agric. Food Chem. 66 (28), 7384–7392. 10.1021/acs.jafc.8b02166 29895146

[B28] Li H. Wu S. Chen J. Wang B. Shi N. (2013). Effect of glutathione depletion on Nrf2/ARE activation by deltamethrin in PC12 Cells. Arh Hig. Rada Toksikol 64 (1), 87–97. 10.2478/10004-1254-64-2013-2251 23585199

[B29] Lin Y. Liu J. Xiang P. Lin P. Ye G. Da Sternberg L. J. B. (2006). Tannin dynamics of propagules and leaves of Kandelia candel and Bruguiera gymnorrhiza in the Jiulong river estuary, Fujian, China. Biogeochemistry 78 (3), 343–359. 10.1007/s10533-005-4427-5

[B30] Liu Y. Alookaran J. J. Rhoads J. M. (2018). Probiotics in autoimmune and inflammatory disorders. Nutrients 10 (10), 1537. 10.3390/nu10101537 PMC6213508 30340338

[B31] Liu J. Luo D. Wu Y. Gao C. Lin G. Chen J. (2019a). The protective effect of sonneratia apetala fruit extract on acetaminophen-induced liver injury in mice. Evid Based Complement Alternat Med. 2019, 6919834. 10.1155/2019/6919834 31320915 PMC6607706

[B32] Liu L. Liang L. Liang H. Wang M. Lu B. Xue M. (2019b). Fusobacterium nucleatum aggravates the progression of colitis by regulating M1 macrophage polarization *via* AKT2 pathway. Front. Immunol. 10, 1324. 10.3389/fimmu.2019.01324 31249571 PMC6582778

[B33] Lopes de Oliveira G. A. Alarcon de la Lastra C. Rosillo M. A. Castejon Martinez M. L. Sanchez-Hidalgo M. Rolim Medeiros J. V. (2019). Preventive effect of bergenin against the development of TNBS-induced acute colitis in rats is associated with inflammatory mediators inhibition and NLRP3/ASC inflammasome signaling pathways. Chem. Biol. Interact. 297, 25–33. 10.1016/j.cbi.2018.10.020 30365937

[B34] Lu M. C. Ji J. A. Jiang Y. L. Chen Z. Y. Yuan Z. W. You Q. D. (2016). An inhibitor of the Keap1-Nrf2 protein-protein interaction protects NCM460 colonic cells and alleviates experimental colitis. Sci. Rep. 6, 26585. 10.1038/srep26585 27215610 PMC4877580

[B35] Ma X. Hu Y. Li X. Zheng X. Wang Y. Zhang J. (2018). Periplaneta americana ameliorates dextran sulfate sodium-induced ulcerative colitis in rats by Keap1/Nrf-2 activation, intestinal barrier function, and gut microbiota regulation. Front. Pharmacol. 9, 944. 10.3389/fphar.2018.00944 30186174 PMC6113651

[B36] Mahmud I. Zilani M. N. H. Biswas N. N. Bokshi B. (2017). Bioactivities of Bruguiera gymnorrhiza and profiling of its bioactive polyphenols by HPLC-DAD. Clin. Phytoscience 3 (1), 1–11. 10.1186/s40816-017-0048-5

[B37] Mo Z. Z. Lin Z. X. Su Z. R. Zheng L. Li H. L. Xie J. H. (2018). Angelica sinensis supercritical fluid CO2 extract attenuates D-Galactose-induced liver and kidney impairment in mice by suppressing oxidative stress and inflammation. J. Med. Food 21 (9), 887–898. 10.1089/jmf.2017.4061 30109956

[B38] Nagalingam N. A. Kao J. Y. Young V. B. (2011). Microbial ecology of the murine gut associated with the development of dextran sodium sulfate-induced colitis. Inflammation Bowel Dis. 17 (4), 917–926. 10.1002/ibd.21462 PMC3058753 21391286

[B39] Nikkhah-Bodaghi M. Maleki I. Agah S. Hekmatdoost A. (2019). Zingiber officinale and oxidative stress in patients with ulcerative colitis: A randomized, placebo-controlled, clinical trial. Complement Ther. Med. 43, 1–6. 10.1016/j.ctim.2018.12.021 30935515

[B40] Nopara B. (1999). Bioactive substances from the mangrove resource. Songklanakarin J. Sci. Technol. 21(3).

[B41] Qu C. Yuan Z. W. Yu X. T. Huang Y. F. Yang G. H. Chen J. N. (2017). Patchouli alcohol ameliorates dextran sodium sulfate-induced experimental colitis and suppresses tryptophan catabolism. Pharmacol. Res. 121, 70–82. 10.1016/j.phrs.2017.04.017 28456683

[B42] Rezaie A. Parker R. D. Abdollahi M. (2007). Oxidative stress and pathogenesis of inflammatory bowel disease: an epiphenomenon or the cause? Dig Dis. Sci. 52 (9), 2015–2021. 10.1007/s10620-006-9622-2 17404859

[B43] Roy S. Madhumita R. Pramanick P. Nayak B. Mitra A. (2018). Antimicrobial activity and phytochemical constituents of Bruguiera gymnorrhiza fruit collected from Indian Sundarbans, the designated World Heritage Site. Int. J. Green herbal Chem. 7 (2), 119–125. 10.24214/ijghc/hc/7/2/11925

[B44] Russo L. Schneider G. Gardiner M. H. Lanes S. Streck P. Rosen S. (2014). Role of pharmacoepidemiology studies in addressing pharmacovigilance questions: a case example of pancreatitis risk among ulcerative colitis patients using mesalazine. Eur. J. Clin. Pharmacol. 70 (6), 709. 10.1007/s00228-014-1660-7 24609467 PMC4025187

[B45] Russo M. Coppola V. Giannetti E. Buonavolontà R. Piscitelli A. Staiano A. (2018). Oral administration of tannins and flavonoids in children with acute diarrhea: a pilot, randomized, control-case study. Ital. J. Pediatr. 44 (1), 64. 10.1186/s13052-018-0497-6 29866147 PMC5987560

[B46] Samman F. S. Elaidy S. M. Essawy S. S. Hassan M. S. (2018). New insights on the modulatory roles of metformin or alpha-lipoic acid versus their combination in dextran sulfate sodium-induced chronic colitis in rats. Pharmacol. Rep. 70 (3), 488–496. 10.1016/j.pharep.2017.11.015 29653414

[B47] Sann H. Erichsen J. Hessmann M. Pahl A. Hoffmeyer A. (2013). Efficacy of drugs used in the treatment of IBD and combinations thereof in acute DSS-induced colitis in mice. Life Sci. 92 (12), 708–718. 10.1016/j.lfs.2013.01.028 23399699

[B48] Sarkar R. Barik R. Biswas P. Sharma A. Karmakar S. Sen T. (2013). Pharmacological studies on bruguiera gymnorrhiza-a focus on dual inhibition of Cox and 5-Lox. Indian J. Pharmacol. 45, S117–S118. 10.4103/0253-7613.182890 PMC4900005 27298502

[B49] Sekhar K. R. Crooks P. A. Sonar V. N. Friedman D. B. Chan J. Y. Meredith M. J. (2003). NADPH oxidase activity is essential for Keap1/Nrf2-mediated induction of GCLC in response to 2-indol-3-yl-methylenequinuclidin-3-ols. Cancer Res. 63 (17), 5636–5645. 10.1097/00130404-200309000-00013 14500406

[B50] Sha T. Igaki K. Yamasaki M. Watanabe T. Tsuchimori N. (2013). Establishment and validation of a new semi-chronic dextran sulfate sodium-induced model of colitis in mice. Int. Immunopharmacol 15 (1), 23–29. 10.1016/j.intimp.2012.10.022 23142502

[B51] Shi C. Zhao X. Liu Z. Meng R. Chen X. Guo N. (2016). Antimicrobial, antioxidant, and antitumor activity of epsilon-poly-L-lysine and citral, alone or in combination. Food Nutr. Res. 60, 31891. 10.3402/fnr.v60.31891 27312785 PMC4911418

[B52] Sobhani I. Tap J. Roudot-Thoraval F. Roperch J. P. Letulle S. Langella P. (2011). Microbial dysbiosis in colorectal cancer (CRC) patients. PloS One 6 (1), e16393. 10.1371/journal.pone.0016393 21297998 PMC3029306

[B53] Song C. H. Kim N. Lee S. M. Nam R. H. Choi S. I. Kang S. R. (2019). Effects of 17beta-estradiol on colorectal cancer development after azoxymethane/dextran sulfate sodium treatment of ovariectomized mice. Biochem. Pharmacol. 164, 139–151. 10.1016/j.bcp.2019.04.011 30981879

[B54] Sudirman S. Agoes N. Jacoeb M. (2014). Proximate compositions, bioactive compounds and antioxidant activity from large-leafed mangrove (Bruguiera gymnorrhiza) fruit. Int. Food Res. J. 21 (6), 2387–2391.

[B55] Tewari R. Rout P. Misra L. N. (2017). Simultaneous RP-HPLC-PDA-RI separation and quantification of pinitol content in Sesbania bispinosa vis-à-vis harvesting age. Plant Biosyst. 151 (5), 924–930. 10.1080/11263504.2016.1265612

[B56] Trivedi P. Mytton J. Evison F. Kamarajah S. K. Reece J. Iqbal T. (2018). A nationwide population-based evaluation of mortality and cancer-risk in patients with ulcerative colitis/primary sclerosing cholangitis—young age at diagnosis and the unmet need to reduce mortality. J. Hepatology 68, S220–S221. 10.1016/s0168-8278(18)30657-3

[B57] Wu X. Wang L. Tang L. Wang L. Cao S. Wu Q. (2018). Salvianolic acid B alters the gut microbiota and mitigates colitis severity and associated inflammation. J. Funct. Foods 46, 312–319. 10.1016/j.jff.2018.04.068

[B58] Yasukawa K. Hirago A. Yamada K. Tun X. Ohkuma K. Utsumi H. (2019). In vivo redox imaging of dextran sodium sulfate-induced colitis in mice using Overhauser-enhanced magnetic resonance imaging. Free Radic. Biol. Med. 136, 1–11. 10.1016/j.freeradbiomed.2019.03.025 30928473

[B59] Zhang X. J. Yuan Z. W. Qu C. Yu X. T. Huang T. Chen P. V. (2018). Palmatine ameliorated murine colitis by suppressing tryptophan metabolism and regulating gut microbiota. Pharmacol. Res. 137, 34–46. 10.1016/j.phrs.2018.09.010 30243842

[B60] Zhou L. Y. Liu D. Y. Xie Y. Yao X. J. Li Y. (2019). Bifidobacterium infantis induces protective colonic PD-L1 and foxp3 regulatory T cells in an acute murine experimental model of inflammatory bowel disease. Gut Liver 13 (4), 430–439. 10.5009/gnl18316 30600673 PMC6622561

