# Supplementary material for: Protective Effect of Bruguiera gymnorrhiza (L.) Lam. Fruit on Dextran Sulfate Sodium-Induced Ulcerative Colitis in Mice: Role of Keap1/Nrf2 Pathway and Gut Microbiota
Source: Front Pharmacol. 2020 Feb 3;10:1602. doi: 10.3389/fphar.2019.01602 (PMC7008401; doi:10.3389/fphar.2019.01602)
Supplement: Supplementary file 1 [file Table_1.docx]

Supplementary Material

**Figure S1** ^1^H NMR spectrum of BGF aqueous extract in DMSO.

**Figure S2** ^13^C NMR spectrum of BGF aqueous extract in DMSO.


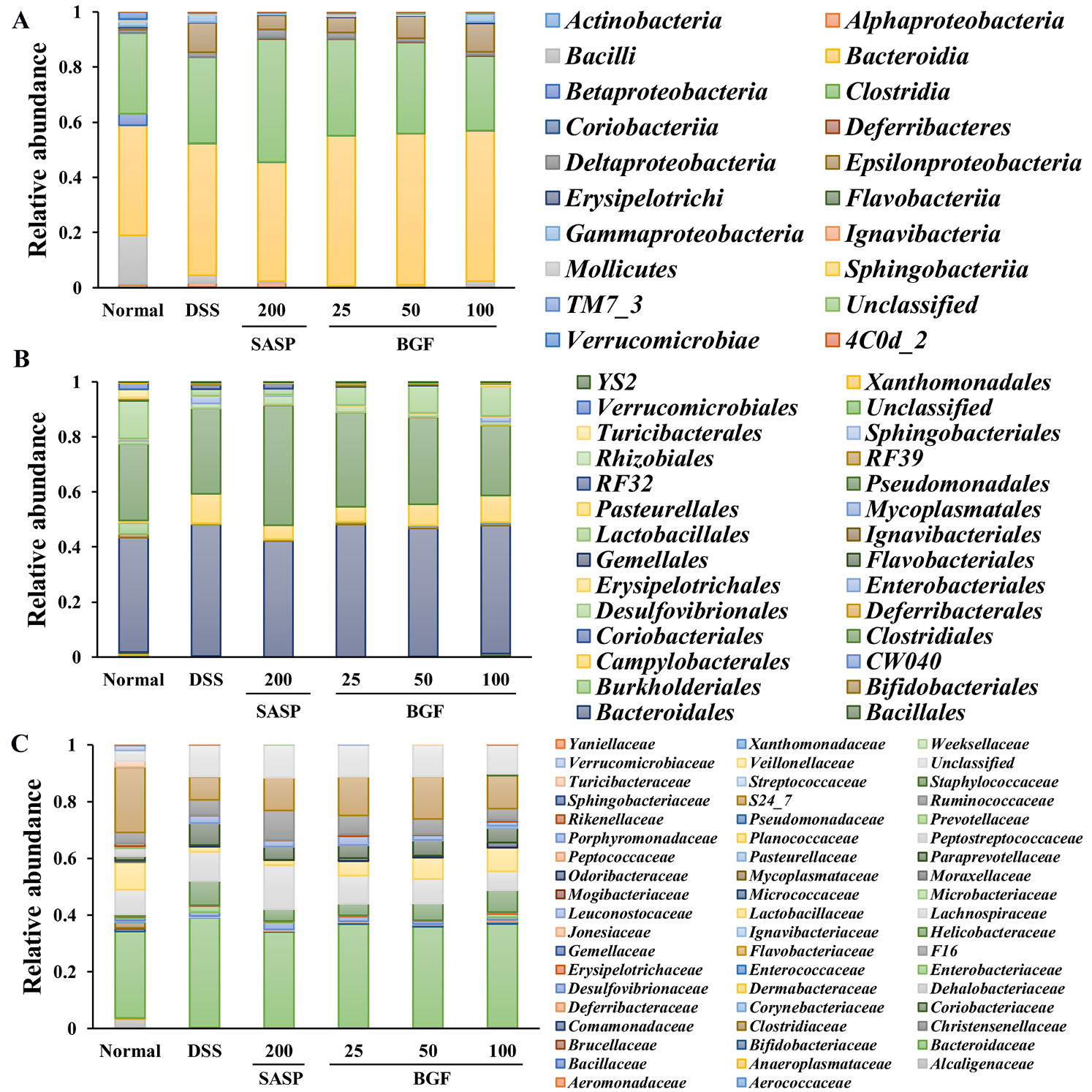


**Figure S3** Effect of BGF on the composition of gut microbiota at the genus level in in normal, SASP, BGL (25 mg/kg of BGF), BGM (50 mg/kg of BGF) and BGH (100 mg/kg of BGF) groups. 16S rDNA gene sequencing analysis of the gut microbiota of mice was shown at the class level **(A)**, at the order level **(B)** and at the family level **(C)**.
